# Supplementary material for: Deep phenotyping and lifetime trajectories reveal limited effects of longevity regulators on the aging process in C57BL/6J mice
Source: Nat Commun. 2022 Nov 11;13:6830. doi: 10.1038/s41467-022-34515-y (PMC9652467; doi:10.1038/s41467-022-34515-y)
Supplement: Supplementary file 1 — Supplementary Information [file 41467_2022_34515_MOESM1_ESM.pdf]

Deep Phenotyping and Lifetime Trajectories Reveal Limited Effects of Longevity Regulators on the Aging Process in C57BL/6J Mice

Kan Xie[1], Helmut Fuchs[2], Enzo Scifo[1], Dan Liu[3], Ahmad Aziz[3,4], Juan Antonio Aguilar-Pimentel[2], Oana Veronica Amarie[2], Lore Becker[2], Patricia da Silva-Buttkus[2], Julia Calzada-Wack[2], Yi-Li Cho[2], Yushuang Deng[1], A. Cole Edwards[1], Lillian Garrett[2,5], Christina Georgopoulou[1], Raffaele Gerlini[2], Sabine M. Hölter[2,5], Tanja Klein-Rodewald[2], Michael Kramer[6], Stefanie Leuchtenberger[2], Dimitra Lountzi[1], Phillip Mayer-Kuckuk[2], Lena L. Nover[1], Manuela A. Oestereicher[2], Clemens Overkott[1], Brandon L. Pearson[1,$], Birgit Rathkolb[2,7,8], Jan Rozman[2,7,§], Jenny Russ[9], Kristina Schaaf[1], Nadine Spielmann[2], Adrián Sanz-Moreno[2], Claudia Stoeger[2], Irina Treise[2], Daniele Bano[10], Dirk H. Busch[11], Jochen Graw[5], Martin Klingenspor[12], Thomas Klopstock[13,14,15], Beverly A. Mock[16], Paolo Salomoni[9], Carsten Schmidt-Weber[17], Marco Weiergräber[18], Eckhard Wolf[8], Wolfgang Wurst[5,14,19], Valérie Gailus-Durner[2], Monique M.B. Breteler[3,20], Martin Hrabě de Angelis[2,7,21,#], Dan Ehninger[1,#]

Affiliations

[1]Translational Biogerontology Lab, German Center for Neurodegenerative Diseases (DZNE), Venusberg-Campus 1/99, 53127 Bonn, Germany.
[2] Institute of Experimental Genetics, German Mouse Clinic, Helmholtz Zentrum München, German Research Center for Environmental Health, 85764 Neuherberg, Germany.
[3]Population Health Sciences, German Center for Neurodegenerative Diseases (DZNE), Venusberg-Campus 1/99, 53127 Bonn, Germany.
[4]Department of Neurology, Faculty of Medicine, University of Bonn, Bonn, Germany
[5]Institute of Developmental Genetics, Helmholtz Zentrum München, German Research Center for Environmental Health, 85764 Neuherberg, Germany.
[6]GEMoaB GmbH, Tatzberg 47, 01307 Dresden, Germany.
[7]Member of German Center for Diabetes Research (DZD), 85764 Neuherberg, Germany.
[8]Institute of Molecular Animal Breeding and Biotechnology, Gene Center, Ludwig-Maximilians-University Munich, Munich, Germany.
[9]Nuclear Function Lab, German Center for Neurodegenerative Diseases (DZNE), Venusberg-Campus 1/99, 53127 Bonn, Germany.
[10]Aging and Neurodegeneration Lab, German Center for Neurodegenerative Diseases (DZNE), Venusberg-Campus 1/99, 53127 Bonn, Germany.
[11]Institute for Medical Microbiology, Immunology, and Hygiene, Technische Universität München, 81675 Munich, Germany.
[12]Molecular Nutritional Medicine, Else Kröner-Fresenius Center, Technische Universität München, 85350 Freising-Weihenstephan, Germany.
[13]Friedrich-Baur-Institut, Department of Neurology, Ludwig-Maximilians-University Munich, 80336 Munich, Germany.
[14]DZNE, German Center for Neurodegenerative Diseases, 80336 Munich, Germany.
[15]Munich Cluster for Systems Neurology (SyNergy), 80336 Munich, Germany.
[16]Laboratory of Cancer Biology and Genetics, CCR, NCI, NIH, Bethesda, MD 20892, USA.

[17]Center of Allergy & Environment (ZAUM), Technische Universität München, and Helmholtz Zentrum München, 85764 Neuherberg, Germany.
[18]Research Group Experimental Neuropsychopharmacology, Federal Institute for Drugs and Medical Devices, 53175 Bonn, Germany.
[19]Chair of Developmental Genetics, TUM School of Life Sciences (SoLS), Technische Universität München, Freising, Germany.
[20]Institute for Medical Biometry, Informatics and Epidemiology, Faculty of Medicine, University of Bonn, Venusberg-Campus 1, 53127 Bonn, Germany.
[21]Chair of Experimental Genetics, TUM School of Life Sciences (SoLS), Technische Universität München, 85354 Freising, Germany.

[$]Current address: Mailman School of Public Health, Columbia University, 630 W. 168th St., New York, NY 10032, USA
[§] Current address: Institute of Molecular Genetics of the Czech Academy of Sciences, Czech Centre for Phenogenomics, Prumyslova 595, Vestec, Czech Republic, 252 50
[#]Equal contribution

Correspondence to: Dan.Ehninger@dzne.de

Supplementary Results

We sought to extract from our dataset ASPs sensitive to PAAI-mediated amelioration specifically in the old group (but not the young group) by selecting phenotypes with an overall significant main effect of age (on the 2-way ANOVA) and a significant difference on the posthoc test between the old intervention group and the old control group, but not on the comparison young intervention group vs. young control group (see **Supplementary Data 6,7,9** for full information on results from statistical analyses which these analyses are based upon). This would be ASPs corresponding to the “rate effect model” introduced in **Fig. 1b**.

The analysis of our *Ghrhr*$^{lit/lit}$ dataset revealed that 7.3% of all ASPs (corresponding to 7 ASPs) followed this pattern (i.e., showed a significant difference between mutant and control in old but not young mice) (**Supplementary Fig. 7a**). Statistical comparison of *Ghrhr*$^{lit/lit}$ effect sizes in young vs. old mice also identified one of these ASPs as significantly different between age groups (activity of Alkaline Phosphatase in the blood plasma; **Supplementary Fig. 7b**).

In the case of our *mTOR*$^{KI/KI}$ cohort, 15.4% of all ASPs (corresponding to 18 ASPs) showed a significant effect of genotype in the old but not the young group based on the posthoc tests (**Supplementary Fig. 7a**). The effect size plot in **Supplementary Fig. 7c** examines how this subset of ASPs was influenced by genotype in the old vs. the young group. This analysis confirms that, based on statistical comparison of Cohen's d effect sizes, several ASPs were differentially ameliorated by *mTOR*$^{KI/KI}$ genotype in the old vs. the young group ($p<0.05$; hemoglobin, hematocrit, plasma triglyceride concentration, subpopulations of CD4+ T cells). However, many of these ASPs appeared to show similar effect sizes in the young vs. the old group of animals (**Supplementary Fig. 7c**). Intraclass correlation analyses of effect sizes in young vs. old mice for this set of ASPs revealed an overall significant correlation (ICC=0.44, p=0.01; **Supplementary Fig. 7c**), suggesting that our strategy to extract ASPs of interest (i.e., ASPs selectively ameliorated in old mice) based on the pattern of posthoc results may generate some false positives.

The analysis of our IF cohort revealed that 22.5% of all ASPs (corresponding to 23 ASPs) followed this pattern (**Supplementary Fig. 7a**). Several of these ASPs were also corroborated by comparison of effect sizes in young vs. old mice, such as plasma insulin concentration, plasma urea concentration, respiratory exchange ratio and the abundance of NKT cells (**Supplementary Fig. 7d**). However, we again noted that in a number of cases diet effect sizes appeared to be similar in young and old mice (despite the posthoc test not revealing a difference between the young IF and young control group upon selection of these ASPs) with an overall significant intraclass correlation of diet effect sizes in young vs. old mice in this set of ASPs (ICC=0.49; p= 0.01; **Supplementary Fig. 7d**).

In conclusion, while these analyses were able to identify ASPs whose selective amelioration in the old group of mice is convincing (see examples discussed above; see also yellow datapoints in effect size plots shown in **Supplementary Fig. 7b-d**), it also suggested some ASPs that are likely false positives (given that effect sizes in the young group were similar to those in the old group). Based on these analyses, the upper bound of our estimate of ASPs following the pattern of selective amelioration in the old group is the one shown in **Supplementary Fig. 8a**. A lower bound may be derived from the number of ASPs with a significant effect size difference between young and old mice (i.e., the yellow datapoints in **Supplementary Fig. 7b-d**); this would suggest that about 1% of all ASPs in the *Ghrhr*$^{lit/lit}$ dataset, 4.3% of all ASPs in the *mTOR*$^{KI/KI}$ cohort and 5.9% of all ASPs in the IF dataset correspond to ASPs selectively ameliorated in old mice but not young mice (i.e., ASPs corresponding to the “rate effect model” introduced in **Fig. 1b**).

Supplementary Discussion

Our analyses generated a large dataset on phenotypes associated with *Ghrhr* loss of function in mice. Novel findings in *Ghrhr$^{lit/lit}$* mice included, for instance, a higher auditory sensitivity, reduced visual acuity as well as an electrocardiographic shortening of the PR interval that may predispose for arrhythmias. In other cases, we confirmed previously reported effects of growth hormone deficiency, such as reduced bone mineral density [1], higher nociceptive sensitivity [2], as well as changes in body composition and metabolism [3], which we found across age groups in *Ghrhr$^{lit/lit}$* mice. Our observation of reduced activity levels in young and old *Ghrhr$^{lit/lit}$* mice, notable across different assays employed (open field, SHIRPA and metabolic phenotyping) is in contrast to a prior report of increased locomotor activity in *Ghrh* (encoding growth hormone releasing hormone) mutant mice [4].

Previous work had established that hypomorphic mTOR mutant mice feature a ca. 20% extension of median lifespan which was associated with a reduced incidence of neoplastic diseases in the mutants [5]. Lifespan studies using the oral mTOR inhibitor rapamycin in mice had yielded median lifespan extensions ranging from 4-26%, depending on dose, age at onset of treatment, sex and site of investigation [6-9]. A large number of the phenotypic effects we observed in mTOR mutants were similar to effects seen under chronic treatment with the pharmacological mTOR inhibitor rapamycin [10,11]: For instance, both the genetic and pharmacological manipulations were associated with age-independent increases in exploratory locomotor activity, red blood cell counts, naïve $CD4^+$- and $CD8^+$-T-cell counts as well as age-independent decreases in hepatic microgranulomas, bronchus-associated lymphatic tissue and unsaturated iron binding capacity. Moreover, both were also associated with a prevention of age-related cardiac hypertrophy and a reduced cancer incidence in old mice and shared adverse effects, such as testicular degeneration, impaired glucose tolerance and an exacerbation of the age-related decrease in NK cells.

However, we also noted a number of effects seen in the mTOR mutants, which we did not observe in mice under chronic rapamycin treatment [10]. For instance, while the specific rapamycin treatment approach we employed previously [10] did not have consistent effects on body and organ weights across treatment cohorts (heart, liver, spleen, brain, kidney; an exception was testis with dramatically reduced weights due to testicular degeneration), the mTOR mutant allele led to clear reductions in body mass, organ weights (brain, heart, kidney, liver, lung, muscle, pancreas, spleen and testis) and reduced retinal thickness. Additional phenotypic effects restricted to the mTOR mutants included a protection against age-related glomerular pathology and elevations in white blood cell and platelet counts. While some of these differential effects may be a matter of rapamycin dosage (e.g., body weight reductions were also seen with higher rapamycin doses [8]), others may not (e.g., chronic oral rapamycin was associated with renal toxicity [10]; mTOR mutants, in contrast, were protected against age-related glomerular pathology and showed no signs of renal toxicity). One limitation of the hypomorphic mTOR mutant mouse model is that it is associated with some degree of embryonic lethality [5,12,13]. Advantages, relative to (oral) pharmacological approaches, include the specific targeting of mTOR (due to the genetic nature of the manipulation) as well as the fact that mTOR inhibition is independent of food intake (which typically declines in old mice).

Supplementary Table 1. Antibodies used in flow cytometry based analyses or applied in the lymphocyte proliferation assay

Panel	Fluorochrome	Cell surface marker	Clone	Company	Dilution
FACS panel 1	FITC	CD11c	HL3	BD Pharmingen, #557400	1:100
	PE	NK1.1	PK136	BD Pharmingen, #553165	1:200
	PE	NKp46	29A1.4	eBioscience, #12-3351-82	1:200
	PE-CF594	CD3e	145-2C11	BD Horizon, #562332	1:100
	PerCP Cy5.5	Ly6C	HK1.4	eBioscience, #45-5932-82	1:400
	PECy7	CD19	1D3	BD Pharmingen, #552854	1:1000
	APC	CD5	53-7.3	BD Pharmingen, #550035	1:2000
	Alexa Fluor 700	CD45	30-F11	BioLegend, #103128	1:1000
	APC-A750	B220	RA3-6B2	Life Technologies, #RM2627	1:100
	PacBlue	CD11b	M1/70.15	Life Technologies, #RM2828	1:800
	PO	Gr1	RB6-8C5	Life Technologies, #RM3030	1:1000
FACS panel 2	PE-CF594	Ly6C	AL-21	BD Horizon, #562728	1:200
	PerCP Cy5.5	CD4	RM4-5	TONBO Biosciences, #65-0042-U025	1:1000
	PECy7	CD62L	MEL-14	eBioscience, #25-0621-82	1:2000
	APC	CD25	PC61	BD Pharmingen, #557192	1:100
	Alexa Fluor 700	CD45	30-F11	BioLegend, #103128	1:1000
	APC-A750	CD8a	5H10	Life Technologies, #MCD0827	1:400
	eF450	CD5	53-7.3	eBioscience, #48-0051-82	1:1000
	bv570	CD44	IM7	BioLegend, #103037	1:100
LPA	Unconjugated	CD3	17A2	eBioscience, #16-0032-86	1 µg/ml
	Unconjugated	CD40	HM40-3	eBioscience, #16-0402-86	1 µg/ml

APC = allophycocyanin; Cy7 = cyanine-7; FACS = fluorescence activated cell sorting; FITC = fluorescein-5-isothiocyanate; LPA = lymphocyte proliferation assay; PE = phycoerythrin; PerCP = peridin chlorophyll; PO = pacific orange

Supplementary Table 2. Molecular assays to study putative drivers of aging

Aging hallmark	Subcategory	Target	Method
**Altered intercellular communication**	**Lipid hormone**	Cox1	WB
	**Inflammation**	*Ccl2*	qPCR
		Ifng	qPCR
		Il1b	qPCR
		Il4	qPCR
		Il6	qPCR
		Il10	qPCR
		Il13	qPCR
		Tnf	qPCR
**Cellular senescence**	**Senescence markers**	*Cdkn2a/p16Ink4a*	qPCR
		Cdkn2a/p19Arf	qPCR
		Cdkn1a/p21	qPCR
	**Tumor suppressor**	*Trp53*	qPCR
**Deregulated nutrient sensing**	**IGF1-signaling**	Igf1	WB
	**mTOR-signaling**	mTOR	WB
		p-4Ebp1 (T37/46)/4Ebp1	WB
		total 4Ebp1	WB
		p-Rps6 (S240/244)/Rps6	WB
		total Rps6	WB
		p-Akt (S473)/Akt	WB
		total Akt	WB
**Genomic instability**	**DNA damage**	8-oxo-guanosine	ELISA
		p-H2ax (S139)/H2ax	WB
		total H2ax	WB
		Tp53bp1	WB
	**Transposons**	*LINE*	qPCR
		L1 5'UTR	qPCR
		L1 3'UTR	qPCR
		MusD	qPCR
		B1	qPCR
		B2	qPCR
**Loss of proteostasis**	**Autophagy**	Atg3	WB
		Atg5	WB
		Lc3a/b II/I	WB
		total Lc3a/b	WB
	**Chaperones**	Hsp60	WB
		Hsp70	WB
		Hsp90	WB
	**Proteasome activity**	20S activity	activity assay
	**Ubiquitin**	Mono-ubiquitin	WB
		Poly-ubiquitin	WB
**Mitochondrial dysfunction**	**Lipid peroxidation**	TBA reactive species	chemical reaction
	**Mitochondrial integrity**	Citrate synthase	WB
		Cox IV	WB
		Sod2	WB
	**Oxidative stress**	ROS production	chemical reaction
		Nitrotyrosine	WB
**Reduced cell proliferation**	**Cell cycle regulators**	*Ccna1*	qPCR
		Ccna2	qPCR
		Ccnb1	qPCR
		Ccnb2	qPCR
		Ccnb3	qPCR
		Ccnc	qPCR
		Ccnd1	qPCR
		Ccnd2	qPCR
		Ccnd3	qPCR
		Ccne1	qPCR
		Ccne2	qPCR
	**Cell proliferation marker**	*Mki67*	qPCR

Akt = Protein kinase B; Cox1 = Cyclooxygenase 1; Cox IV = Cytochrom c oxidase IV; ELISA = Enzyme-linked immunosorbent assay; Hsp = Heat shock protein; Igf1 = Insulin-like growth factor 1; Lc3 = Microtubule associated protein 1A/1B light chain 3; mTOR = Mechanistic target of rapamycin; qPCR = Quantitative polymerase chain reaction; ROS = Reactive oxygen species; Rps6 = Ribosomal protein S6; Sod2 = Superoxide dismutase 2; TBA = Thiobarbituric acid; Tp53bp1 = Tumor suppressor p53-binding protein 1; WB = Western blot

Supplementary Table 3. Primer sequences used for real-time quantitative PCR analyses

Aging hallmark	Gene/transposon	Primer forward	Primer reverse
**Altered intercellular communication**	*Ccl2*	AAGAGATCAGGGAGTTTGCT	CTGCCTCCATCAACCACTTT
	Ifng	CTTTGGACCCTCTGACTTGAG	TCAATGACTGTGCCGTGG
	Ilb1	GAAGAAGAGCCCATCCTCTG	TCATCTCGGAGCCTGTAGTG
	Il4	GCATTTTGAACGAGGTCACAG	TGGAAGCCCTACAGACGAG
	Iil6	AGTCCGGAGAGGAGACTTCA	ATTTCCACGATTTCCCAGAG
	Il10	AGCCGGGAAGACAATAACTG	GGAGTCGGTTAGCAGTATGTTG
	Il13	ACCAAAATCGAAGTAGCCCAC	GCAAAGTCTGATGTGAGAAAGG
	Tnf	CTTCTGTCTACTGAACTTCGGG	CAGGCTTGTCACTCGAATTTTG
**Cellular proliferation**	*Ccna1*	GGGTGTTGACTGAAAATGAGC	CACGTTTGGCTGGTTCATTG
	Ccna2	GTCCTTGCTTTTGACTTGGC	ACGGGTCAGCATCTATCAAAC
	Ccnb1	CTGACCCAAACCTCTGTAGTG	CCTGTATTAGCCAGTCAATGAGG
	Ccnb2	CCTCAGAACACCAAAGTACCAG	CCTTCATGGAGACATCCTCAG
	Ccnb3	TCCAGTGCTATCATGCCAAG	CTGTCACTGTCATCCTGTATGG
	Ccnc	GCATTTGTATCAGGGCAAGC	GAAACTTTAGGTCCTTTTGGCG
	Ccnd1	GCCCTCCGTATCTTACTTCAAG	GCGGTCCAGGTAGTTCATG
	Ccnd2	GTGTTCCTATTTCAAGTGCGTG	AGCCAAGAAACGGTCCAG
	Ccnd3	GCGTGCAAAAGGAGATCAAG	GATCCAGGTAGTTCATAGCCAG
	Ccne1	GCGAGGATGAGAGCAGTTC	AAGTCCTGTGCCAAGTAGAAC
	Ccne2	GACGTTCATCCAGATAGCTCAG	TCCCATTCCAAACCTGAAGC
	Mki67	TGCCCGACCCTACAAAATG	GAGCCTGTATCACTCATCTGC
**Cellular senescence**	*Cdkn2a/p16Ink4a*	CCCAACGCCCCGAACT	GCAGAAGAGCTGCTACGTGAA
	Cdkn2a/p19Arf	CTCTGGCTTTCGTGAACATG	TCGAATCTGCACCGTAGTTG
	Cdkn1a/p21	CAGATCCACAGCGATATCCAG	AGAGACAACGGCACACTTTG
	Trp53	ATGTTCCGGGAGCTGAATG	CCCCACTTTCTTGACCATTG
**Genomic instability**	*LINE*	TGAGTGGAACACAACTTCTGC	CAGGCAAGCTCTCTTCTTGC
	L1 5'UTR	CTGCCTTGCAAGAAGAGAGC	AGTGCTGCGTTCTGATGATG
	L1 3'UTR	CCAGCAAACACAGAAGTGGATGCTCA	TTTGCAAGTCCAATGGGCCTCTCT
	MusD	ATAGAGGCCGCTTCTTTGC	TGAGACTCCACCAAATGTCC
	B1	CATGGTGGCGCACGCCTTTAATCC	CCAGGCTGGCCTCGAACTCAGAAA
	B2	GGGCTGGAGAGATGGCTCAGTGGT	GCCACCATGTGGTTGCTGGGAATTG
	Actb	CCCTGAAGTACCCCATTGAAC	CCATGTCGTCCCAGTTGGTAA

Supplementary Table 4. Antibodies used in the context of Western Blot based analyses

Aging hallmark	Target	Antibody used	Dilution
**Altered intercellular communication**	Cox1	Cell Signaling Technologies, #4841, polyclonal	1:2000
**Deregulated nutrient sensing**	Igf1	Abcam, #ab9572, polyclonal	1:1000
	mTOR	Cell Signaling Technologies, #2983, clone 7C10	1:2000
	p-4Ebp1 (T37/46)	Cell Signaling Technologies, #2855, clone 236B4	1:2000
	total 4Ebp1	Cell Signaling Technologies, #9644, clone53H11	1:30000
	p-Rps6 (S240/244)	Cell Signaling Technologies, #2215, polyclonal	1:2000
	total Rps6	Cell Signaling Technologies, #2217, clone 5G10	1:10000
	p-Akt (S473)	Cell Signaling Technologies, #9271, polyclonal	1:2000
	total Akt	Cell Signaling Technologies, #9272, polyclonal	1:5000
**Genomic instability**	p-H2ax (S139)	Cell Signaling Technologies, #2577, polyclonal	1:2000
	H2ax	Cell Signaling Technologies, #2595, polyclonal	1:2000
	Tp53bp1	Abnova, #PAB12506, polyclonal	1:2000
**Loss of proteostasis**	Atg3	Cell Signaling Technologies, #3415, polyclonal	1:2000
	Atg5	Cell Signaling Technologies, #12994, clone D5F5U	1:2000
	Lc3a/b	Cell Signaling Technologies, #12741, clone D3U4C	1:3000
	Hsp60	Cell Signaling Technologies, #4870, clone D307	1:10000
	Hsp70	Cell Signaling Technologies, #4872, polyclonal	1:10000
	Hsp90	Cell Signaling Technologies, #4874, polyclonal	1:10000
	Mono-/Poly-ubiquitin	Thermo Fisher Scientific, #PA5-76144, polyclonal	1:2000
**Mitochondrial dysfunction**	Citrate synthase	Cell Signaling Technologies, #14309, clone D7V8B	1:2000
	Cox IV	Cell Signaling Technologies, #4850, clone 3E11	1:2000
	Sod2	Cell Signaling Technologies, #13194, clone D9V9C	1:2000
	Nitrotyrosine	Enzo Life Science, #BML-SA297-0100, polyclonal	1:2000
	Actin	MP Biomedicals, #SKU 0869100, clone C4	1:30000

Akt = Protein kinase B; Cox1 = Cyclooxygenase 1; Cox IV = Cytochrom c oxidase IV; Hsp = Heat shock protein; Igf-1 = Insulin-like growth factor 1; Lc3 = Microtubule associated protein 1A/1B light chain 3; mTOR = Mechanistic target of rapamycin; Rps6 = Ribosomal protein S6; Sod2 = Superoxide dismutase 2; Tp53bp1 = Tumor suppressor p53-binding protein 1

Supplementary Table 5. Age and genotype effect in *GHRHR*-related (endo)phenotypic measures in humans

The association between *GHRHR* eQTL dosage and each (endo)phenotypic measure was assessed using multiple linear regression models adjusted for age, sex and population stratification using the first ten genetic principal components. Boldface indicates significance. *) Age was mean-centered before inclusion in the regression models.

Determinant	Change in outcome (SD) [estimate (95% CI)]					
	Platelet	*p-value*	*Cholesterol level*	*p-value*	*LDL level*	*p-value*
GHRHR eQTL	***-0.067 (-0.123, -0.011)***	***0.019***	***-0.059 (-0.115, -0.003)***	***0.039***	***-0.076 (-0.133, -0.019)***	***0.009***
*Age**	***-0.007 (-0.013, -0.001)***	***0.026***	***0.019 (0.013, 0.025)***	***$1.5*10^{-09}$***	***0.016 (0.010, 0.022)***	***$5.7*10^{-07}$***
GHRHR eQTL x age	0.000 (-0.004, 0.004)	0.891	-0.003 (-0.007, 0.001)	0.143	-0.003 (-0.008, 0.001)	0.096

CI = confidence interval; eQTL = expression quantitative trait loci; *GHRHR* = growth hormone releasing hormone receptor; LDL = low-density lipoproteins; SD = standard deviation

Supplementary Table 6. Characteristics of the human study population

	Overall (n= 3034)
Age [year], mean ± SD (range)	56.2 ± 14.3 (30 - 95)
Sex, n (%)	
Women	1700 (56)
Men	1334 (44)
MTOR eQTL genotype, n (%)	
GG	1506 (49.6)
CG	1203 (39.7)
CC	281 (9.3)
GHRHR eQTL genotype, n (%)	
GG	254 (8.4)
AG	1339 (44.1)
AA	1388 (45.7)

eQTL = expression quantitative trait locus; *GHRHR* = growth hormone releasing hormone receptor; *MTOR* = mammalian target of rapamycin; SD = standard deviation.
MTOR eQTL genotype: 44 missing; *GHRHR* eQTL genotype: 53 missing

Supplementary Table 7. Age and genotype effect in *MTOR*-related (endo)phenotypic measures in humans

The association between *MTOR* eQTL dosage and each (endo)phenotypic measure was assessed using multiple linear regression models adjusted for age, sex and population stratification using the first ten genetic principal components. Boldface indicates significance. *) Age was mean-centered before inclusion in the regression models.

Determinant	Change in outcome (SD) [estimate (95% CI)]									
	Body fat	*p-value*	*% Body fat*	*p-value*	*Body weight*	*p-value*	*Creatine level*	*p-value*	*MET hours*	*p-value*
MTOR eQTL	***0.066 (0.011, 0.12)***	***0.018***	***0.048 (0.002, 0.095)***	***0.042***	***0.055 (0.007, 0.103)***	***0.024***	***0.079 (0.016, 0.141)***	***0.013***	***0.059 (0, 0.118)***	***0.049***
*Age**	***0.014 (0.01, 0.017)***	**$2.2*10^{-15}$**	***0.022 (0.02, 0.025)***	**$< 2.0*10^{-16}$**	***-0.003 (-0.006, 0)***	***0.033***	***0.011 (0.007, 0.015)***	**$7.3*10^{-08}$**	***-0.013 (-0.016, -0.009)***	**$1.6*10^{-12}$**
MTOR eQTL x age	***-0.005 (-0.008, -0.001)***	***0.021***	***-0.004 (-0.007, 0)***	***0.031***	-0.002 (-0.006, 0.001)	0.149	0.000 (-0.004, 0.005)	0.960	0.801 (-1.400, 3.010)	0.169

CI = confidence interval; eQTL = expression quantitative trait loci; MET = metabolic equivalent of task; mTOR = mammalian target of rapamycin; SD = standard deviation

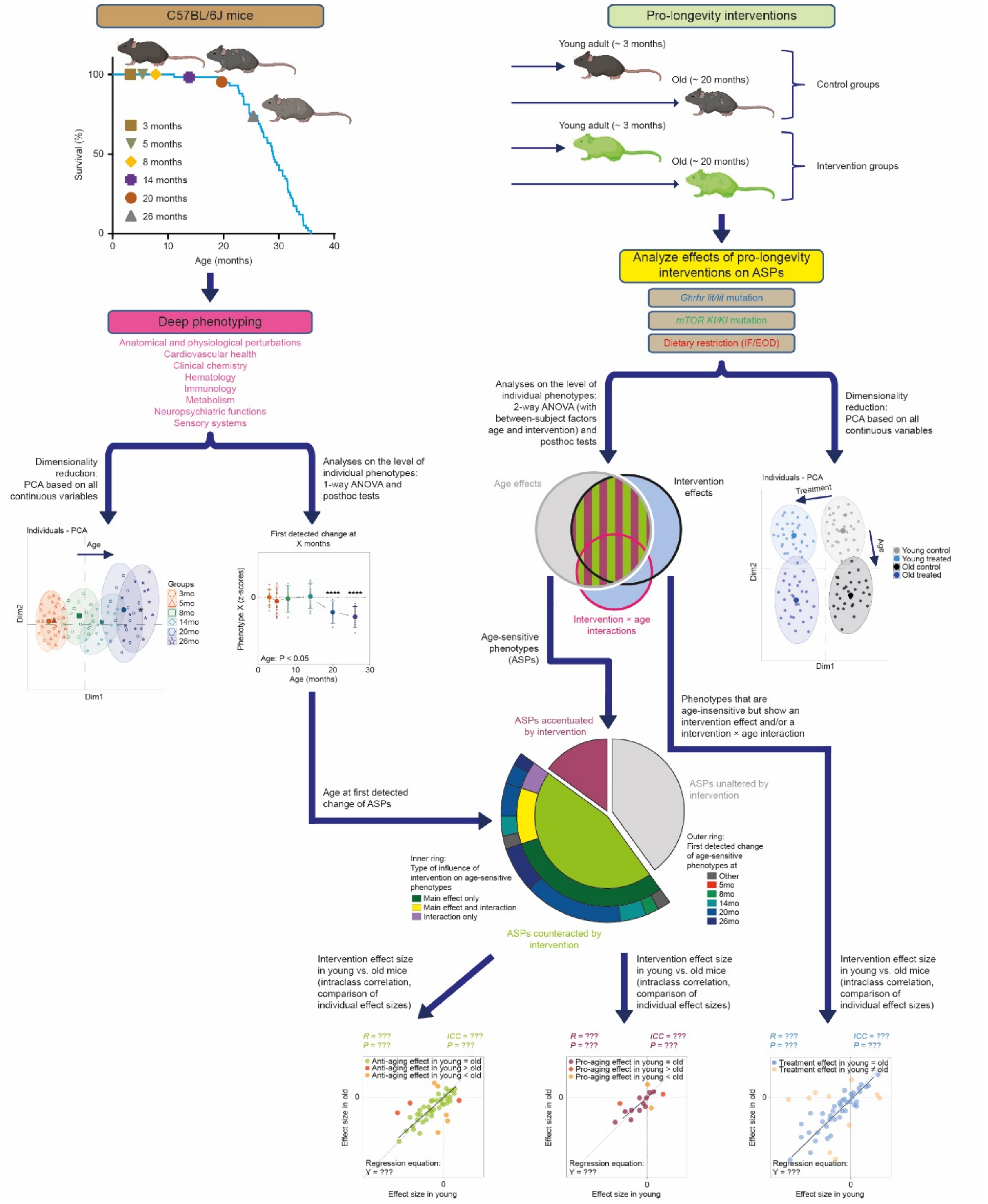

Supplementary Figure 1: Schematic illustration of the analytical workflow of the current study. The figure summarizes our analytical approach. We performed large-scale phenotypic analyses in 3-month, 5-month, 8-month, 14-month, 20-month and 26-month old C57BL/6J mice to identify age-sensitive phenotypes (ASPs) and estimate their aging trajectories. To identify ASPs, we performed one-way ANOVA with the between-subjects factor age (or Kruskal-Wallis test in the case of non-parametric data). For each ASP, we used posthoc analyses to determine at which age phenotypes first differed significantly from the 3-month reference group (results are presented in **Fig. 2a–e** and fully described in **Supplementary Data 1**). We carried out PCA to visualize how these six age groups differed

from each other when extracting the first 2 principal components from this multidimensional dataset (results are presented in **Fig. 2f**).

We examined three pro-longevity interventions for their effects on age-dependent phenotypic change. For each intervention, we carried out large-scale phenotypic analyses using a study design that included a young control group, a young intervention group, an aged control group and an aged intervention group.

To visualize overall age and intervention effects in our multidimensional dataset, we carried out PCA on all continuously distributed phenotypes. We provide, for each animal, the values of the first 2 principal components in a scatter plot (results are presented in **Fig. 3b,6b and 8b;** compare to schematics outlined in **Fig. 1b**).

On the level of individual phenotypes, we used two-way ANOVAs with the between-subject factors age and intervention (or aligned rank transform in the case of non-parametric data) to extract main effects of age, main effects of intervention as well as intervention × age interactions (**Supplementary Fig. 5**; **Supplementary Data 6**, **7** and **9**). These analyses help to differentiate, on the level of individual phenotypes, between the "rate effect" model as well as "combination of rate effect and baseline effect" model on the one hand (**Fig. 1b**, left and middle panels; ASPs with a significant interaction term) and the "baseline effect model" on the other hand (**Fig. 1b**, panels to the right; ASPs without a significant interaction term). We show Venn diagrams featuring the number of phenotypes with main effects and/or an interaction (**Fig. 3c, 6c and 8c**). We further examine phenotypes with a main effect of age (age-sensitive phenotypes, ASPs): Sunburst charts show the proportion of ASPs opposed (effect sizes of age and intervention are in opposing directions), accentuated (effect sizes of age and intervention are in the same direction) or not influenced by an intervention (**Fig. 3c, 6c and 8c**). For ASPs ameliorated by an intervention, the inner circle of the sunburst chart shows the proportion of ASPs that features a significant main effect of intervention and/or a significant intervention × age interaction. The outer circle of the sunburst chart shows at which age changes in the corresponding ASPs were first detected based on data available from our baseline study. We carried out posthoc analyses in an attempt to identify ASPs opposed by intervention only in the old but not in the young group of mice (**Supplementary Fig. 7**); these analyses were meant to identify ASPs consistent with the "rate effect" model (**Fig. 1b**, left panels).

To show how intervention effect sizes in young mice relate to intervention effect sizes in aged mice (overall and on the level of individual phenotypes), we provide effect size plots for different subsets of phenotypes: 1) ASPs countered by intervention (i.e., ASPs with a significant main effect of intervention and/or a significant intervention × age interaction and effect sizes of age and intervention that go in opposing directions; this corresponds to the central green section of the sunburst chart) (**Fig. 3g, 6g and 8g**). 2) ASPs accentuated by intervention (i.e., ASPs with a significant main effect of intervention and/or a significant intervention × age interaction and effect sizes of age and intervention that go in the same direction; this corresponds to the central magenta section of the sunburst chart) (**Fig. 3h, 6h and 8h**). 3) Phenotypes featuring a main effect of intervention and/or an intervention × age interaction but not a main effect of age (**Fig. 3i, 6i and 8i**).

We examined, for each phenotype individually, whether intervention effects differed significantly between young and old mice (phenotypes with significant differences are highlighted in the effect size plots) (**Fig. 3f-i, 6f-i and 8f-i**). These analyses were also used to help differentiate, on the level of individual phenotypes, between the "rate effect" model as well as "combination of rate effect and baseline effect" model on the one hand (**Fig. 1b**, left and middle panels; ASPs with a significant difference in intervention effect size when comparing young vs. old mice) and the "baseline effect model" on the other hand (**Fig. 1b**, ASPs without a significant difference in intervention effect size when comparing young vs. old mice). We performed linear regression to test how well effects in young and old mice are correlated across these sets of phenotypes (**Fig. 3f-i, 6f-i and 8f-i**). Additionally, we performed intraclass correlation analyses which reflect not only the degree of correlation but also the agreement between measures in the young and old group (**Fig. 3f-i, 6f-i and 8f-i**).

Finally, we used linear models to derive standardized coefficients, with their 95% confidence intervals, for age, intervention and intervention × age interaction terms (**Fig. 4**). If there were many phenotypes that followed the “rate effect” model, this would be captured by many phenotypes with interaction term coefficients that are different from zero. If “baseline effects” were the dominant pattern, this would be reflected by many intervention terms being different from zero.
Together, these analyses were performed to help differentiate between the models outlined in **Fig. 1b**.
Figure elements created with BioRender.com.

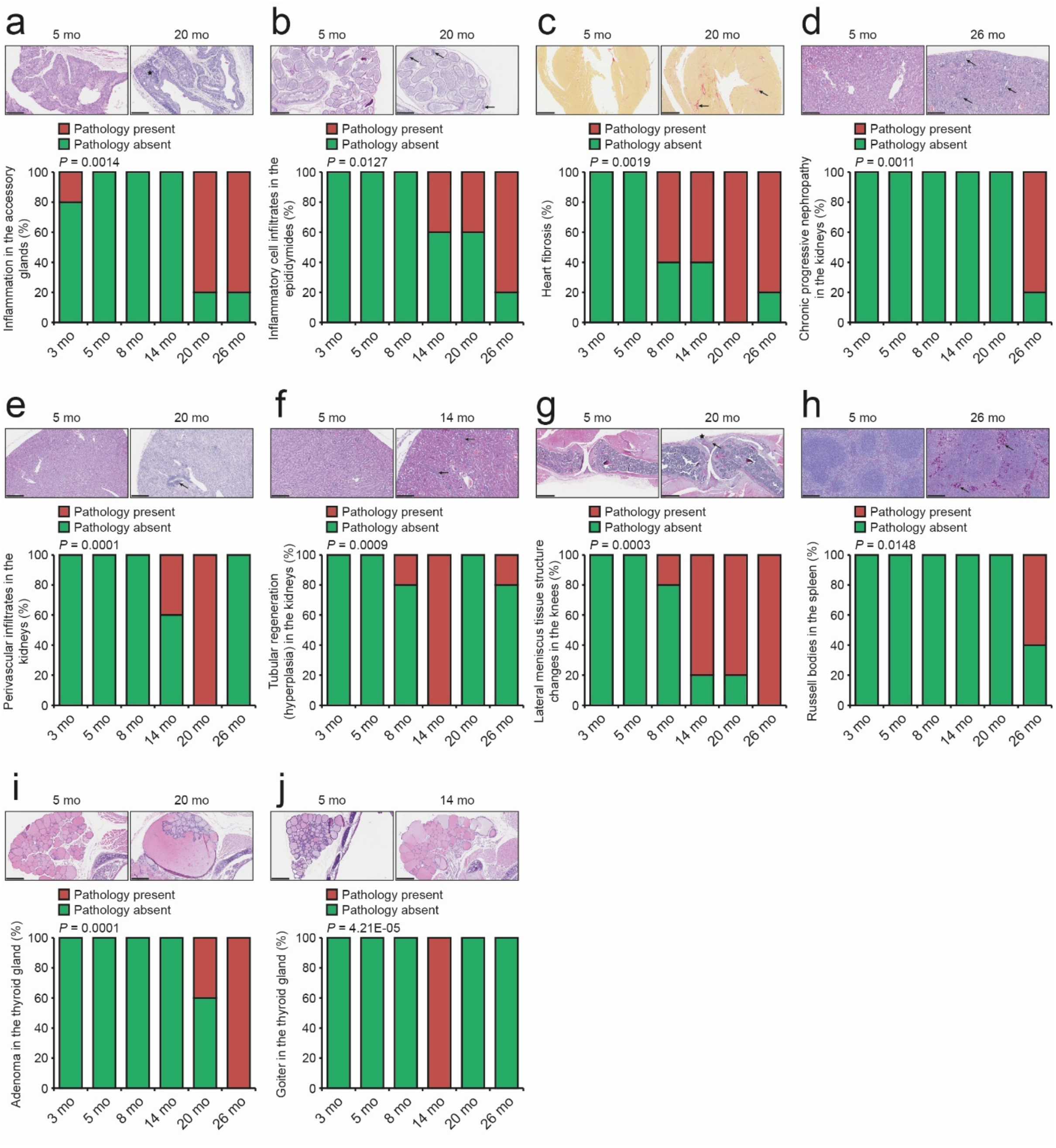

Supplementary Figure 2: Pathological findings in aging C57BL/6J mice. The graphs show the relative proportion of animals in each age group affected by inflammation in the accessory glands (**a**, scale bar: 250 µm), inflammatory infiltrates in the epididymides (**b**, scale bar: 500 µm), heart fibrosis (**c**, scale bar: 1 mm), chronic progressive nephropathy (**d**, scale bar: 250 µm), perivascular infiltrates in the kidneys (**e**, scale bar: 500 µm), tubular regeneration in the kidneys (**f**, scale bar: 250 µm), lateral meniscus tissue structure changes in the knees (**g**, scale bar: 1 mm), Russel bodies in the spleen (**h**, scale bar: 250 µm), adenoma (**i**, scale bar: 250 µm) or goiter (**j**, scale bar: 250 µm) of the thyroid gland. Representative examples of histopathological findings in older mice (alongside healthy tissue in younger mice) are shown in the images accompanying the graphs. Data are based on n=5 mice per age group and were analyzed using two-sided Fisher's exact tests. For further details, see **Supplementary Data 1**.Source data are provided as a Source Data file.

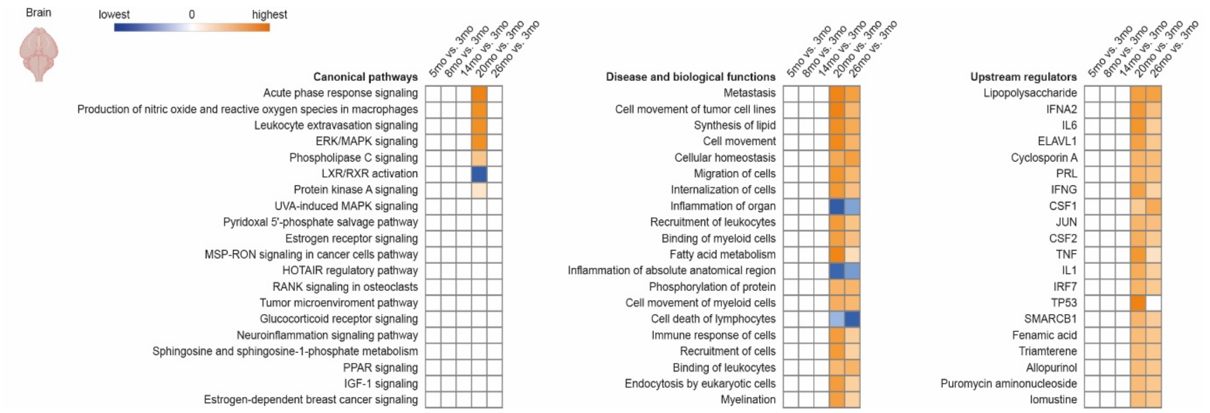

Supplementary Figure 3: RNA-seq-based transcriptome analysis captures gene expression changes in the brain across the lifespan in male C57BL/6J mice. Ingenuity Pathway Analysis shows top canonical pathways, diseases and biological functions as well as predicted upstream regulators of genes differentially expressed in the brain relative to the 3-month old group (FDR<0.05). Positive z-scores (in orange) indicate activating effects, while negative z-scores (in blue) indicate inhibitory effects on corresponding processes. Number of mice per age group: 3 months: n=7; 5 months: n=9; 8 months: n=8; 14 months: n=9; 20 months: n=7; 26 months: n=5. For further details, see **Supplementary Data 2**. Figure elements created with BioRender.com.

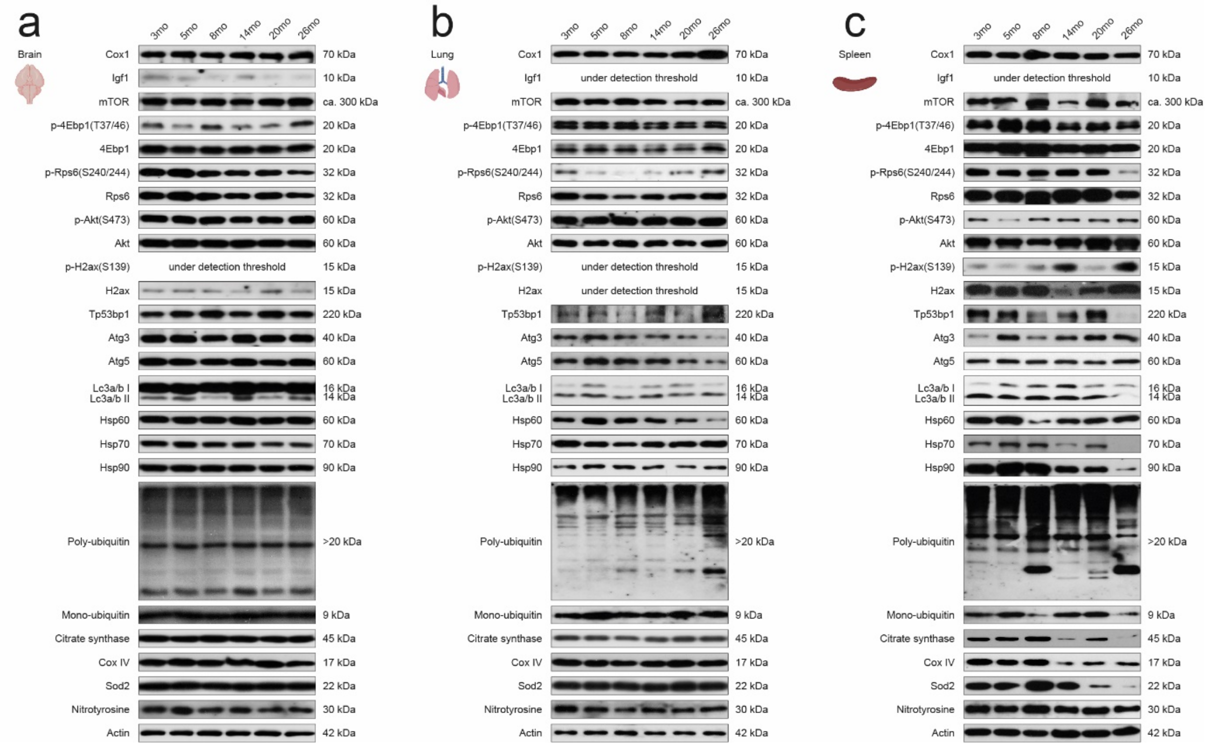

Supplementary Figure 4: Western-blot-based quantification of proteins linked to hallmarks of aging. Representative band densities are shown for proteins detected in brain (**a**), lung (**b**) and spleen (**c**). Individual western blot experiments were performed once, respectively. All samples were derived from the same experiment and, for each marker, gels/blots were processed in parallel with all experimental conditions counterbalanced across gels/blots. The exact sample size for the detection of a given target is presented in **Supplementary Data 5.** Figure elements created with BioRender.com.

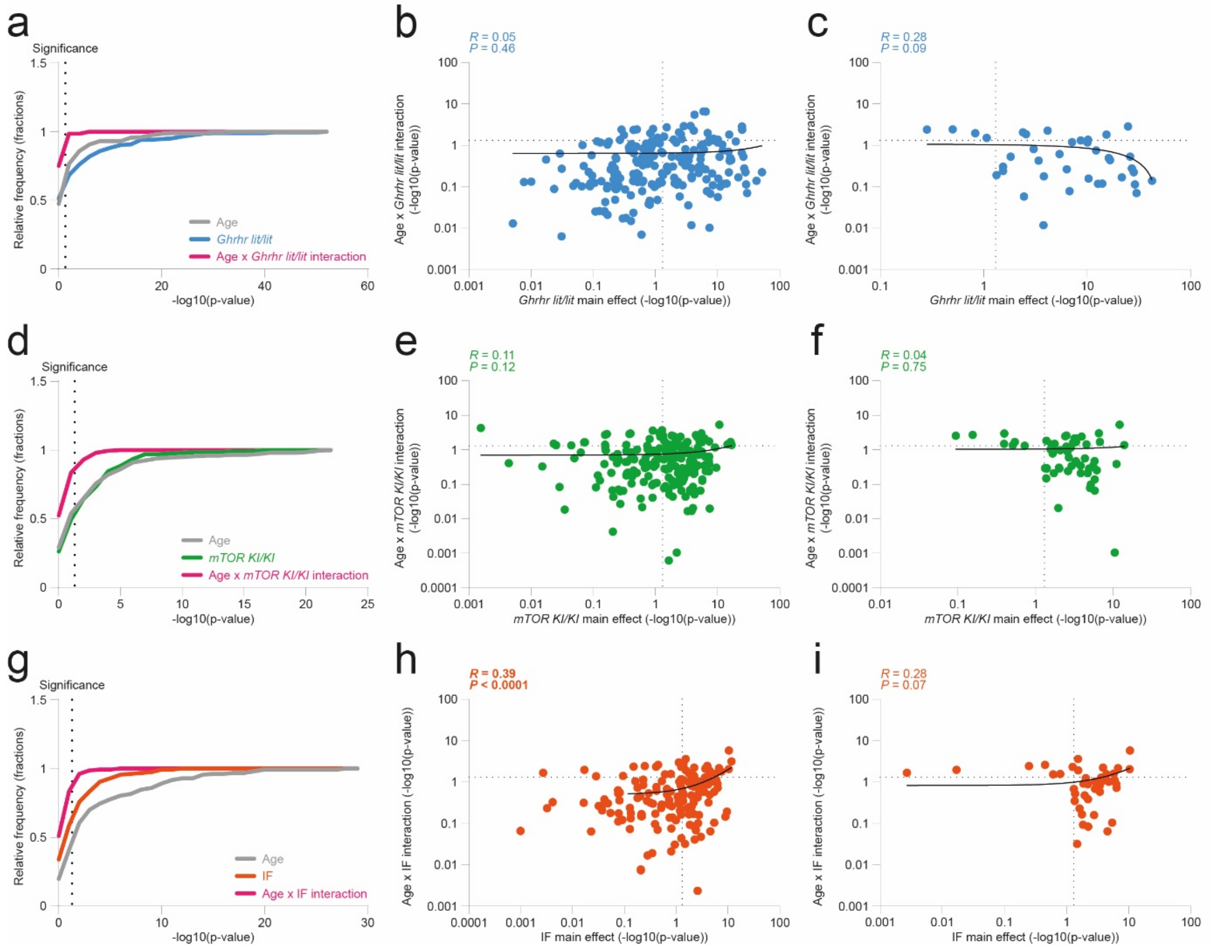

Supplementary Figure 5: PAAIs - systematic analysis of main effects of age, main effects of intervention and intervention × age interactions. a,**d**,**g**: These plots show, for all 3 PAAIs examined in the present paper, cumulative frequencies of -log10(p-values) for age effects, intervention effects and intervention × age interactions for all phenotypes analyzed via two-way ANOVA or aligned rank transform. The vertical dotted line marks the significance threshold ($p<0.05$; corresponding to ~1.3 after the log transformation and multiplication with -1). **b**,**e**,**h**: These scatter plots show, for all PAAIs assessed, -log10(p-values) for intervention main effects plotted vs. -log10(p-values) of intervention × age interactions for all phenotypes analyzed via two-way ANOVA or aligned rank transform. The vertical and horizontal dotted lines mark the significance threshold ($p<0.05$). The graphs also show regression lines, correlation coefficients and p-values derived from linear regression analyses. **c**,**f**,**i**: Scatter plots show, for all PAAIs assessed, -log10(p-values) for intervention main effects plotted vs. -log10(p-values) of intervention × age interactions for age-sensitive phenotypes countered by intervention. The vertical and horizontal dotted lines mark the significance threshold ($p<0.05$). The graphs also show regression lines, correlation coefficients and p-values derived from linear regression analyses. Sample sizes (number of mice per group) underlying these analyses are detailed, for each phenotype and all PAAIs, in **Supplementary Data 6**, **7** and **9**.

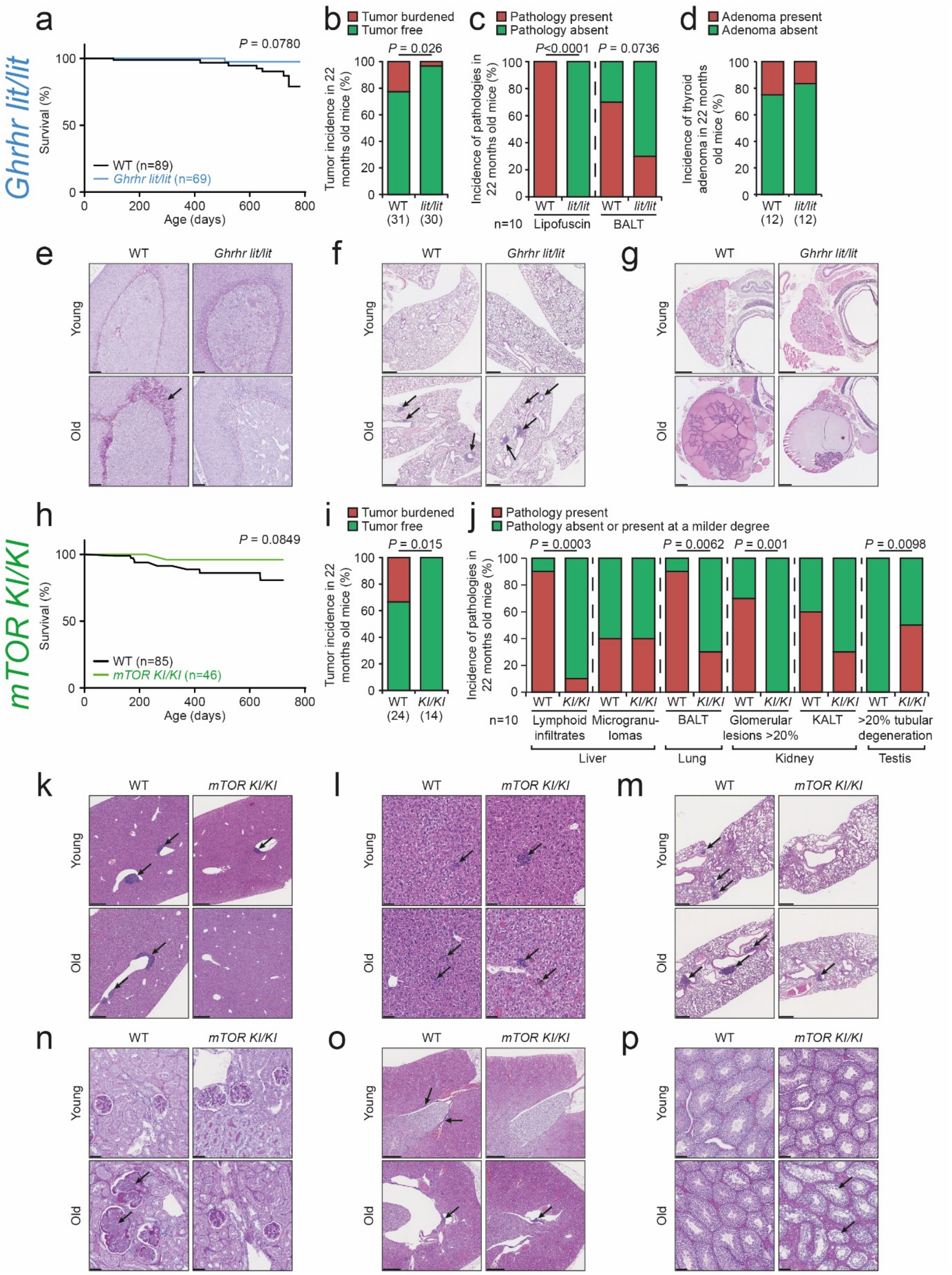

Supplementary Figure 6: Survival and pathological analyses in *Ghrhr*$^{lit/lit}$ and hypomorphic *mTOR*$^{KI/KI}$ mice. The figure shows provisional survival data and summarizes histopathological analyses for aging *Ghrhr*$^{lit/lit}$ (**a-g**) and *mTOR*$^{KI/KI}$ (**h-p**) mice as well as the corresponding WT littermate controls. **a**,**h**: Provisional survival curves were established based on cases of natural deaths in *Ghrhr*$^{lit/lit}$ and *mTOR*$^{KI/KI}$ cohorts aged in our facility (p-

values shown are based on analyses via Log-rank (Mantel-Cox) test). **b-d**, **i-j**: These panels show the percentage of aged mutant vs. WT animals affected by the pathological findings specified in the graphs (p-values are based on analysis via a two-sidedFisher's exact test). *p<0.05, **p<0.01, ***p<0.001, ****p<0.0001. BALT: bronchus-associated lymphoid tissue; KALT: kidney-associated lymphoid tissue. **e-g**, **k-p**: The images show representative examples of histopathological findings in aged mice as well as the corresponding healthy intact tissue in young animals. **e**, Lipofuscin deposits in the adrenal gland; scale bar: 100 µm. **f**, Bronchus-associated lymphoid tissue (BALT); scale bar: 500 µm. **g**, Thyroid gland adenoma; scale bar: 250 µm. **k**, Lymphoid infiltrates in the liver; scale bar: 500 µm. **l**, Microgranulomas in the liver; scale bar: 50 µm. **m**, Bronchus-associated lymphoid tissue (BALT); scale bar: 500 µm. **n**, Glomerular lesions in the kidney; scale bar: 50 µm. **o**, Kidney-associated lymphoid tissue (KALT); scale bar: 500 µm. **p**, Tubular degeneration in the testis; scale bar: 100 µm. Unadjusted p-values are shown. Additional information is available in **Supplementary Data 6** and **7**. Source data are provided as a Source Data file.

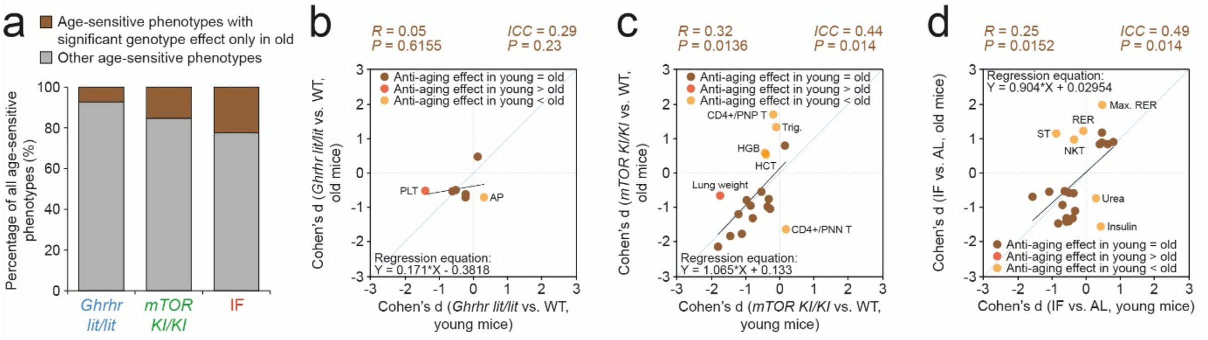

Supplementary Figure 7: Analysis of ASPs sensitive to PAAI-mediated effects specifically in the old groups of mice. a, Percentage of ASPs that feature a significant intervention effect in the old group (posthoc test old invention group vs. old control group, p<0.05), but not the young group of animals (posthoc test young intervention group vs. young control group, p>0.05). **b-d**, Effect size plots show Cohen's d effect sizes of intervention (**b**: *Ghrhr$^{lit/lit}$* vs. WT; **c**: *mTOR$^{KI/KI}$* vs. WT; **d**: IF vs. AL) in the young group vs. the old group of animals. To assess overall relationships between phenotypic intervention effect sizes in young vs. old animals, we performed linear regression (see correlation coefficient R, p-value, linear regression equation; black line: regression line; blue line: line through origin with slope 1) and intraclass correlation (see ICC, p-value) analyses. The graphs also show whether individual phenotypes had significantly different effect sizes in young vs. old mice (phenotypes with significant differences are identified by their abbreviated name; see **Supplementary Data 6, 7** and **9** for full description).

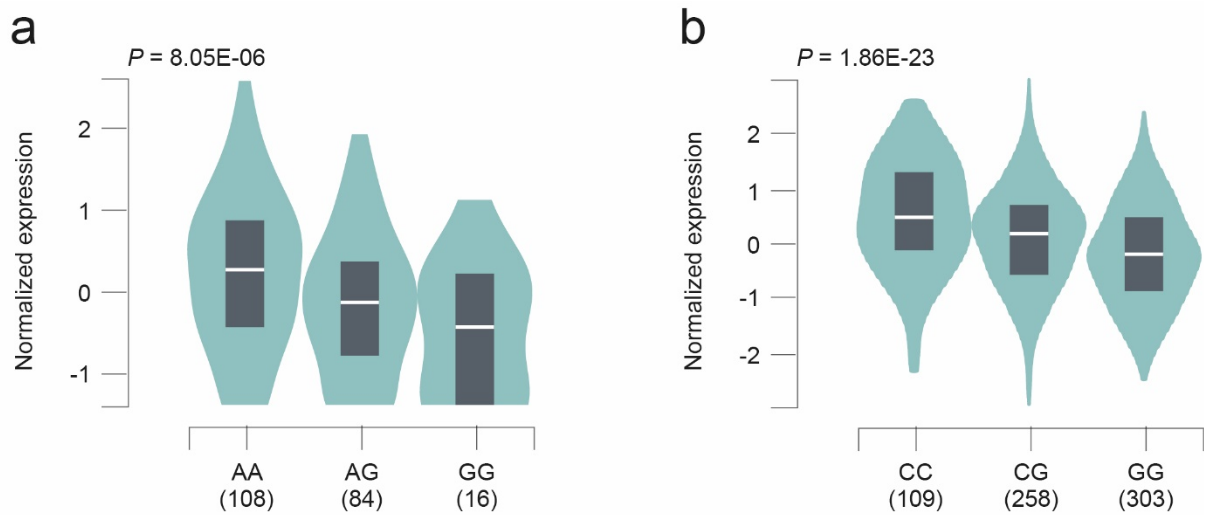

Supplementary Figure 8: Effect sizes of *GHRHR* and *mTOR* eQTLs on gene expression levels. Violin plots of *GHRHR* expression levels (**a**), stratified by the cis-eQTL at SNP rs11772180 (chr7: 30810998_A_G), as well as *MTOR* expression levels (**b**), stratified by the cis-eQTL at SNP rs2295079 (chr1: 11262508_C_G), as obtained from the Genotype Tissue Expression portal (genome build 38). Note that eQTLs for *GHRHR* have thus far only been assessed in human liver tissue. The *MTOR* eQTL has been validated in a wide range of human tissues, including brain, heart, skin, muscle and various gastrointestinal tissues (**b** shows expression levels in blood). The numbers below the horizontal axes indicate the number of samples assessed for each genotype for estimating gene expression levels. The shaded regions represent the density distributions of the samples for each genotype. The box plots indicate the interquartile ranges (black) and the median value (white lines) of gene expression for each genotype. The p-values shown represent the statistical significance of the normalized effect size based on a linear regression model; the normalized effect size is defined as the slope of the linear regression and is computed as the effect of the alternative allele relative to the reference allele in the human genome reference.

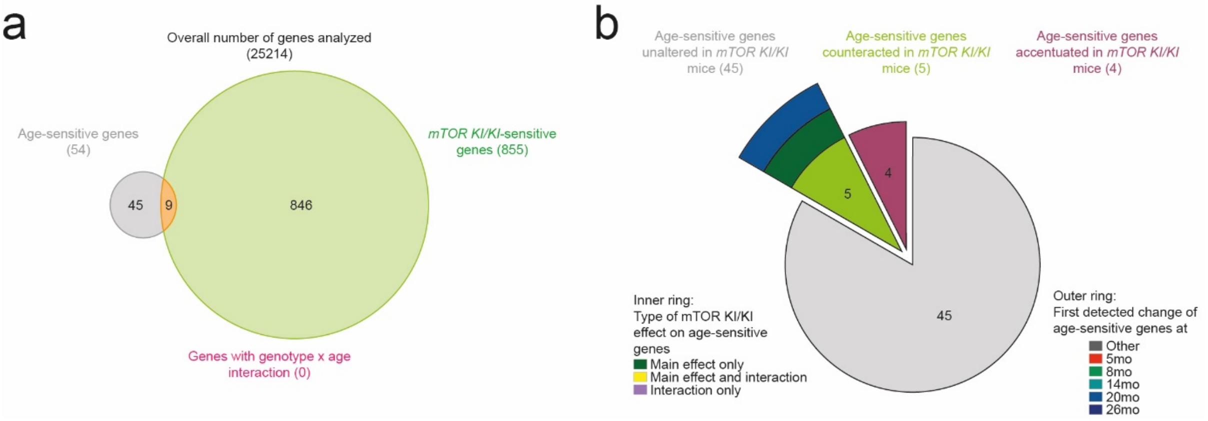

Supplementary Figure 9: RNA-seq-based transcriptome analysis of spleen in young and old *mTOR*$^{KI/KI}$ mice as well as wildtype littermate controls. This figure summarizes the results of an RNA-seq-based differential expression analysis comparing gene expression in the spleen of young and old *mTOR*$^{KI/KI}$ mice as well as WT littermate controls (n=3 per group). **a**, Venn diagram shows the number of age-sensitive genes (FDR<0.05), genotype-sensitive genes (FDR<0.05), genes with an interaction (FDR<0.05) (all derived from DESeq2-based differential expression analyses of RNA-seq data) as well as the intersection of these sets. **b**, Sunburst chart shows the number of age-sensitive genes either unaltered, countered or accentuated by the *mTOR*$^{KI/KI}$ genotype. For age-sensitive genes (ASGs) countered by *mTOR*$^{KI/KI}$ genotype, the inner ring shows the proportion of genes with a main effect of genotype, a genotype × age interaction or both a main effect and an interaction. The outer ring shows when changes in the corresponding ASGs were first detected based on data available from our baseline study.

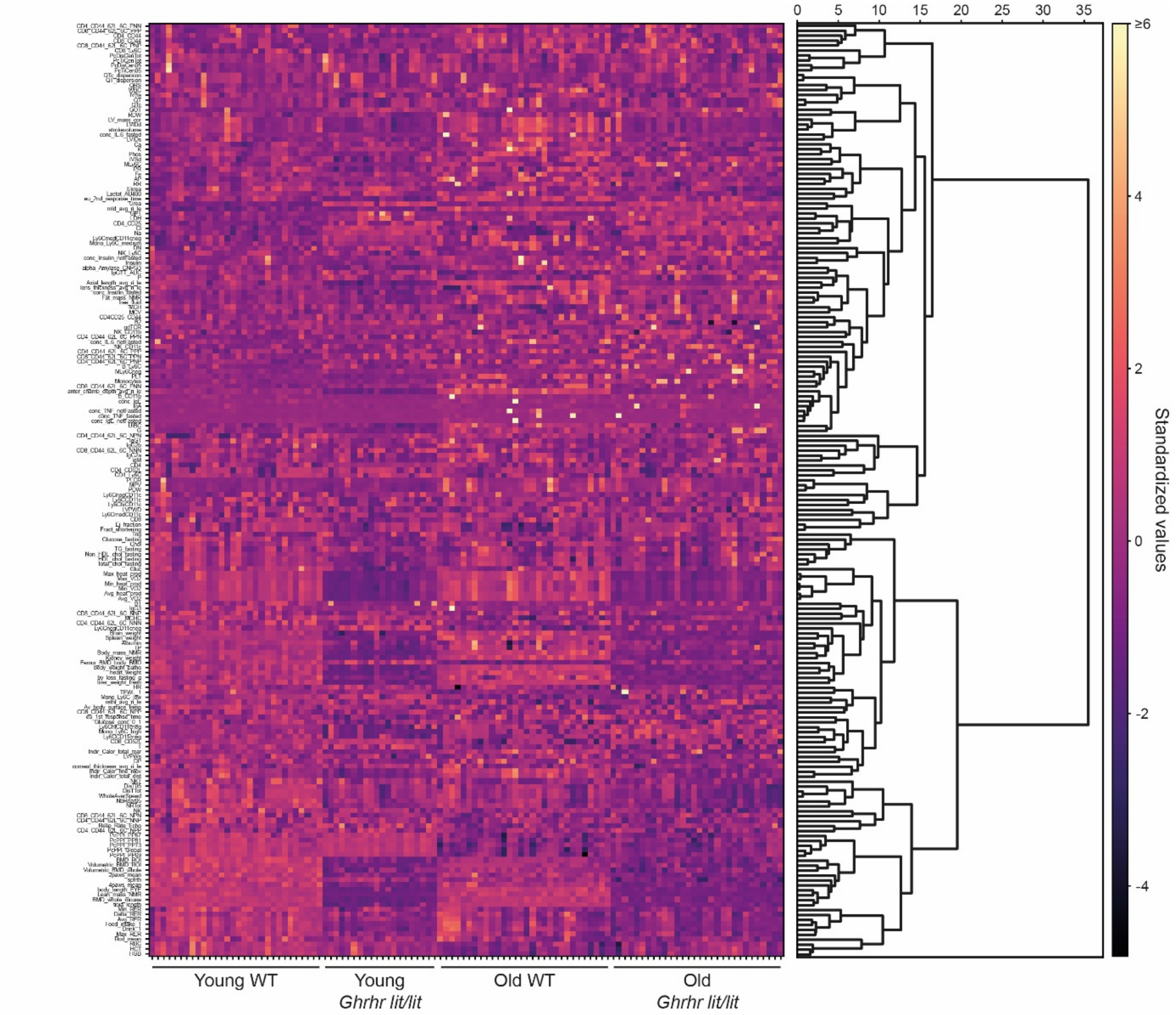

Supplementary Figure 10: Cluster analysis of phenotypes in *Ghrhr*$^{lit/lit}$ cohort. The figure shows results of hierarchical clustering applied to the phenotypic data obtained in the context of the analyses of our *Ghrhr*$^{lit/lit}$ cohort. In order to be able to see what relationships might exist between phenotypes within our young control group, we performed hierarchical clustering on the young WT animals only (hence, yielding phenotype clusters that are independent of age- and genotype-associated phenotypic variation); the resulting clusters and distances between them can be extracted from the dendrogram shown in the figure. The heatmap to the left demonstrates standardized phenotype values for all phenotypes and animals (including young mutant mice and the old groups). How many clusters one identifies depends on the distance at which the dendrogram is cut. Analyses of genotype influences on clusters derived from different ways to cut the dendrogram (based on different minimal inter-cluster distances) are summarized in **Supplementary Data 10**.

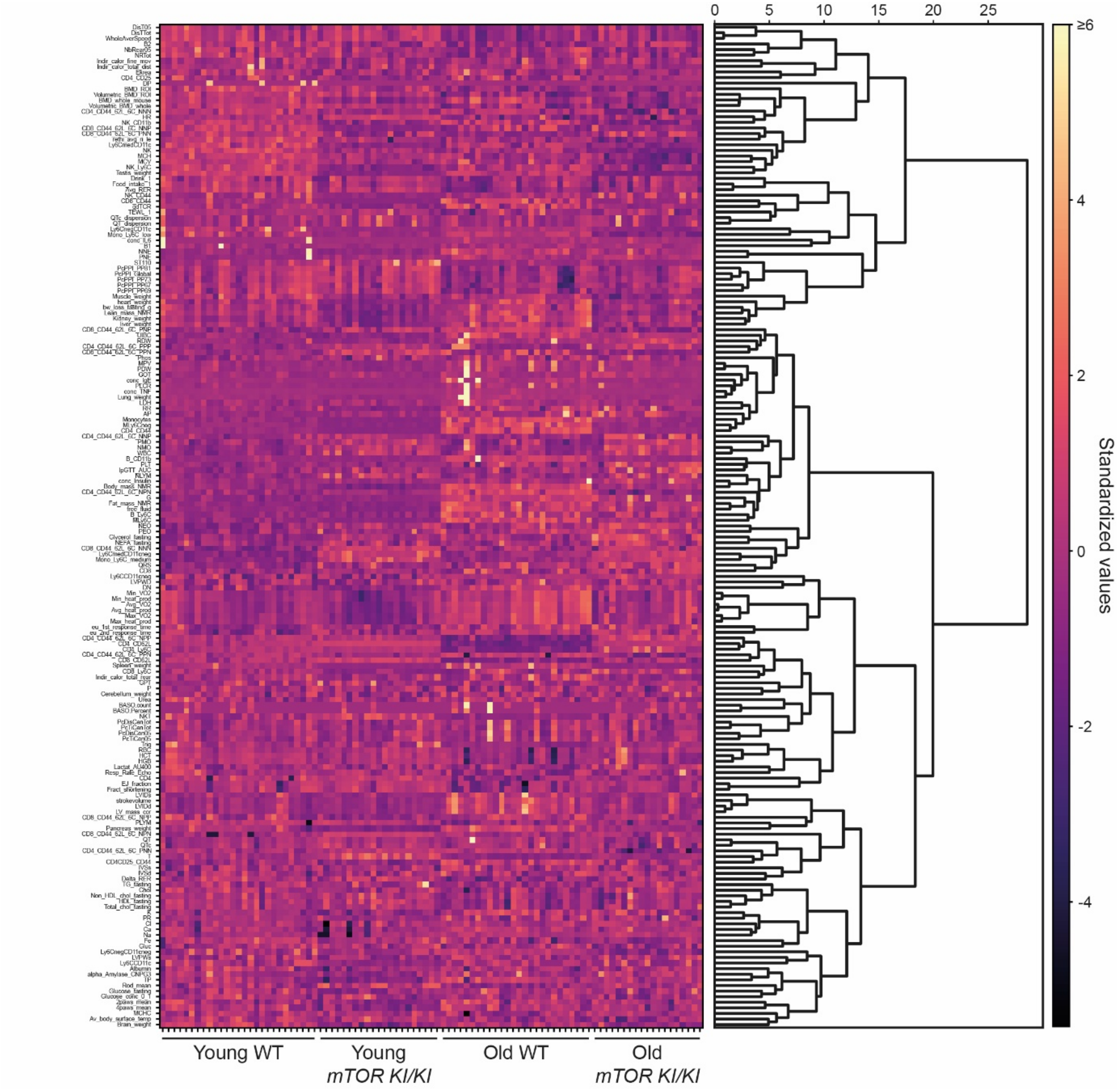

Supplementary Figure 11: Cluster analysis of phenotypes in *mTOR*$^{KI/KI}$ cohort. The figure shows results of hierarchical clustering applied to the phenotypic data obtained from our *mTOR*$^{KI/KI}$ cohort. In order to be able to see what relationships exist between phenotypes within our young control group, we performed hierarchical clustering on the young WT animals only (hence, yielding phenotype clusters that are independent of age- and genotype-associated phenotypic variation); the resulting clusters and distances between them can be extracted from the dendrogram shown in the figure. The heatmap to the left demonstrates standardized phenotype values for all phenotypes and animals (including young mutant mice and the old groups). How many clusters one identifies depends on the distance at which the dendrogram is cut. Analyses of genotype influences on clusters derived from different ways to cut the dendrogram (based on different minimal inter-cluster distances) are summarized in **Supplementary Data 11**.

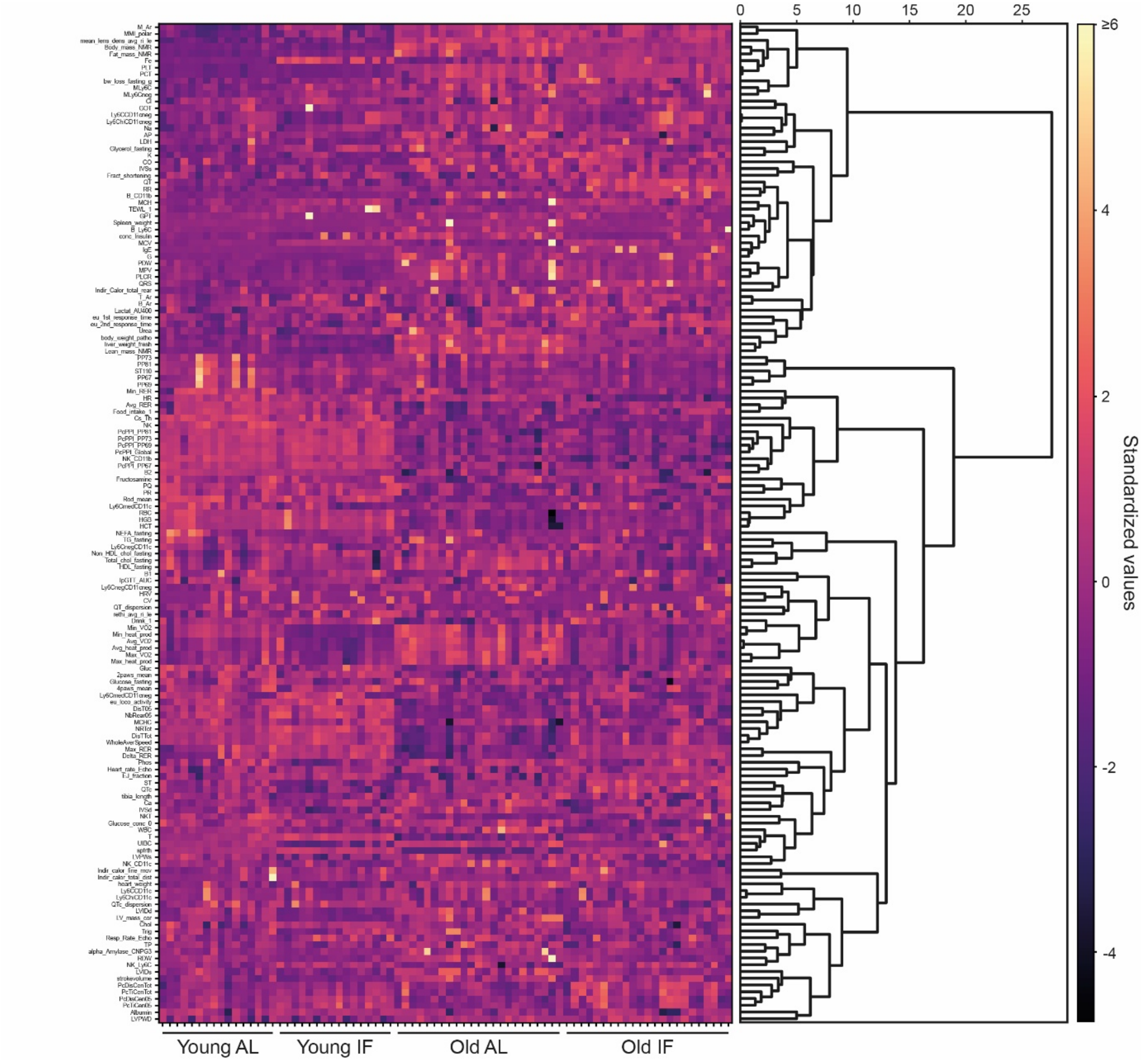

Supplementary Figure 12: Cluster analysis of phenotypes in IF cohort. The figure shows results of hierarchical clustering applied to the phenotypic data obtained from our IF cohort. In order to be able to see what relationships exist between phenotypes within our young control group, we performed hierarchical clustering on the young AL animals only (hence, yielding phenotype clusters that are independent of age- and diet-associated phenotypic variation); the resulting clusters and distances between them can be extracted from the dendrogram shown in the figure. The heatmap to the left demonstrates standardized phenotype values for all phenotypes and animals (including young IF mice and the old groups). How many clusters one identifies depends on the distance at which the dendrogram is cut. Analyses of diet influences on clusters derived from different ways to cut the dendrogram (based on different minimal inter-cluster distances) are summarized in **Supplementary Data 12**.

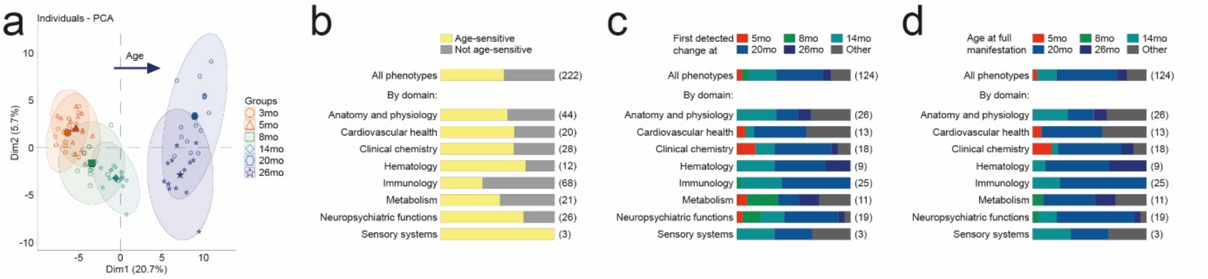

Supplementary Figure 13: Deep phenotyping analyses of age-dependent changes in tumor-free C57BL/6J mice. **a**–**d**, Deep phenotyping results in wildtype tumor-free C57BL/6J mice. **a**, Principal component analysis of deep phenotyping data (number of mice: 3-month old, n=15; 5-month old, n=14; 8-month old, n=15; 14-month old, n=13; 20-month old, n=15; 26-month old, n=13). **b**, Relative proportion of age-sensitive phenotypes among all phenotypes examined. **c**,**d**, Age at first detectable change (**c**) and age at full manifestation (**d**) of age-sensitive phenotypes (ASPs) shown as proportion of all ASPs.

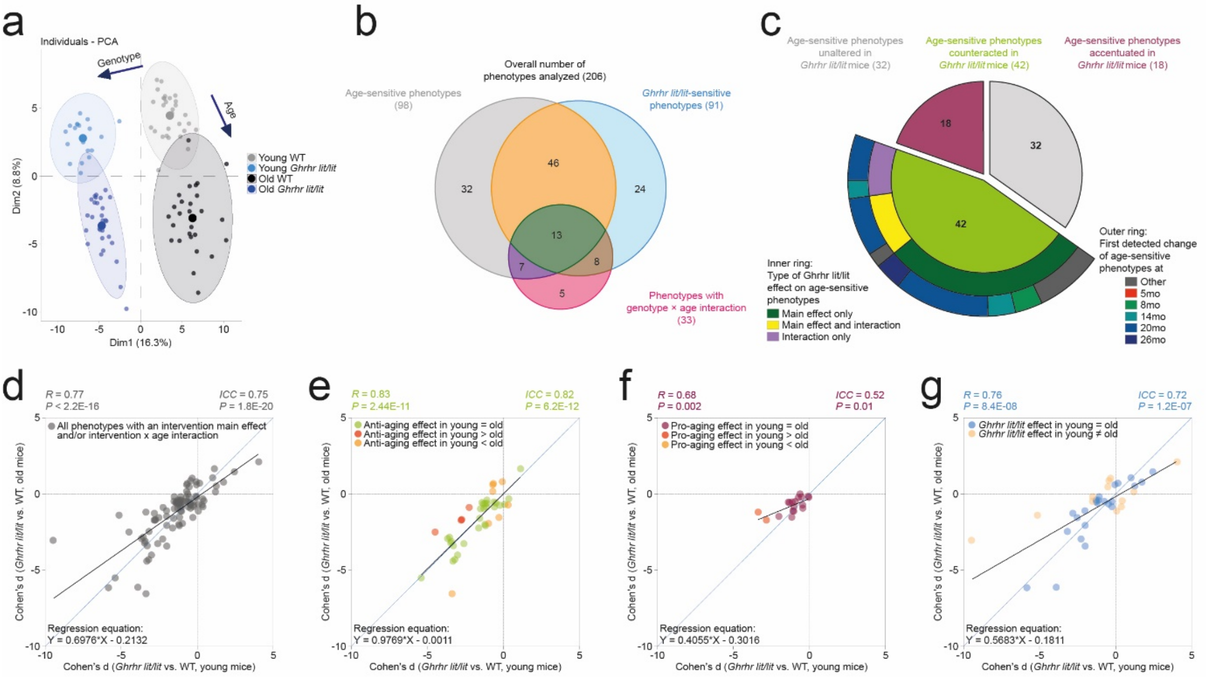

Supplementary Figure 14: Anti-aging effects induced by the *Ghrhr*$^{lit/lit}$ mutation; analysis restricted to tumor-free mice. **a**, Principal component analysis of deep phenotyping data (number of mice: young WT, n=30; young *Ghrhr*$^{lit/lit}$, n=20; old WT, n=25; old *Ghrhr*$^{lit/lit}$, n=29). **b,** Venn diagram shows the number of age-sensitive phenotypes, genotype-sensitive phenotypes, phenotypes with a genotype × age interaction and their intersection. Two-way ANOVAs with the between-subjects factors age and genotype followed by Fisher's LSD posthoc analyses (where appropriate) were used for data analysis. **c**, Sunburst chart shows the number of age-sensitive phenotypes either unaltered, counteracted or accentuated by the *Ghrhr*$^{lit/lit}$ mutation. For age-sensitive phenotypes counteracted by the *Ghrhr*$^{lit/lit}$ mutation, the inner ring shows the proportion of phenotypes with a main effect of genotype, a genotype × age interaction or both a main effect and an interaction. The outer ring shows when changes in the corresponding ASPs were first detected based on data available from our baseline study. **d–g**, Scatter plots show the effect size of *Ghrhr*$^{lit/lit}$ genotype in young mice plotted vs. the effect size of *Ghrhr*$^{lit/lit}$ genotype in old mice for different sets of phenotypes: **e**, ASPs counteracted by genotype via a main effect and/or an interaction (i.e., corresponding to the central green section of the sunburst chart in **c**); **f**, ASPs accentuated by genotype. **g**, Phenotypes featuring a main effect of *Ghrhr*$^{lit/lit}$ genotype and/or a genotype × age interaction but not a main effect of age. **d**, all phenotypes shown in **e-g** collapsed into one panel. ICC = intraclass correlation. Statistical effect size comparisons were performed via two-sided z-tests. Our analyses are based on unadjusted p-values. For further details, see **Supplementary Data 6.**

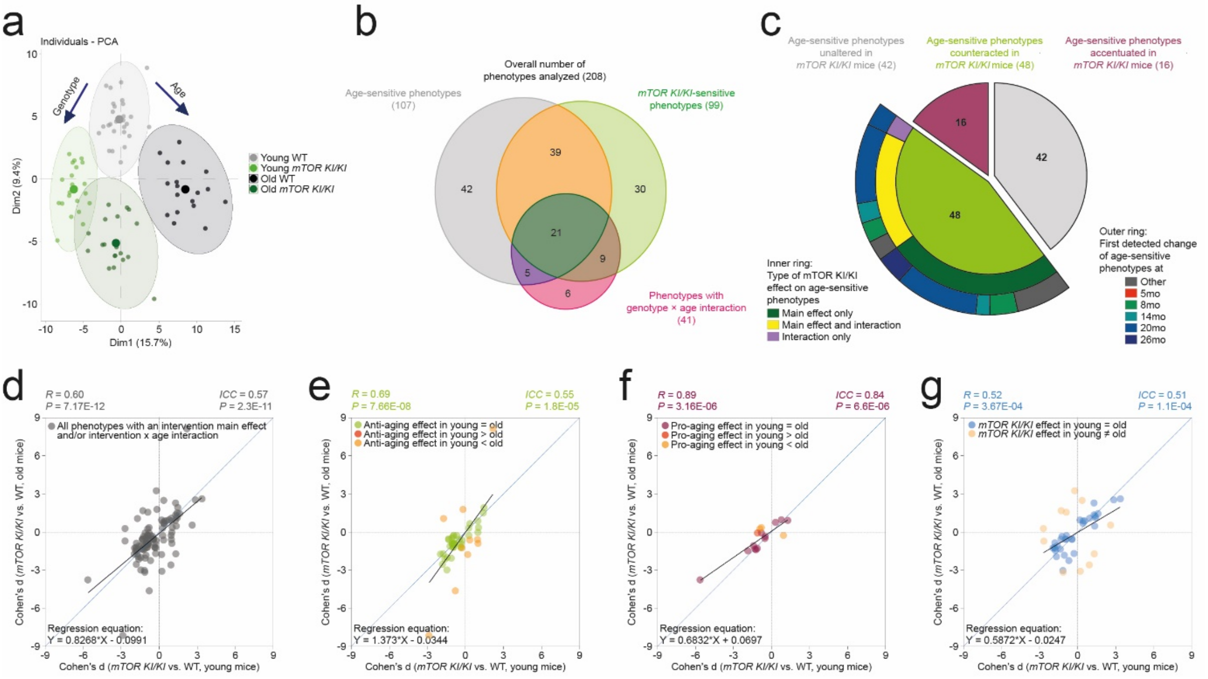

Supplementary Figure 15: Anti-aging effects induced by a hypomorphic mTOR mutation; analysis restricted to tumor-free mice. **a**, Principal component analysis of deep phenotyping data (number of mice: young WT, n=27; young $mTOR^{KI/KI}$, n=21; old WT, n=18; old $mTOR^{KI/KI}$, n=19). **b,** Venn diagram shows the number of age-sensitive phenotypes, genotype-sensitive phenotypes, phenotypes with a genotype × age interaction and their intersection. Two-way ANOVAs with the between-subjects factors age and genotype followed by Fisher's LSD posthoc analyses (where appropriate) were used for data analysis. **c**, Sunburst chart shows the number of age-sensitive phenotypes either unaltered, counteracted or accentuated by the $mTOR^{KI/KI}$ mutation. For age-sensitive phenotypes counteracted by the $mTOR^{KI/KI}$ mutation, the inner ring shows the proportion of phenotypes with a main effect of genotype, a genotype × age interaction or both a main effect and an interaction. The outer ring shows when changes in the corresponding ASPs were first detected based on data available from our baseline study. **d–g**, Scatter plots show the effect size of $mTOR^{KI/KI}$ genotype in young mice plotted vs. the effect size of $mTOR^{KI/KI}$ genotype in old mice for different sets of phenotypes: **e**, ASPs counteracted by genotype via a main effect and/or an interaction (i.e., corresponding to the central green section of the sunburst chart in **c**); **f**, ASPs accentuated by genotype. **g**, Phenotypes featuring a main effect of $mTOR^{KI/KI}$ genotype and/or a genotype × age interaction but not a main effect of age. **d**, all phenotypes shown in **e-g** collapsed into one panel. ICC = intraclass correlation. Statistical effect size comparisons were performed via two-sided z-tests. Our analyses are based on unadjusted p-values. For further details, see **Supplementary Data 7.**

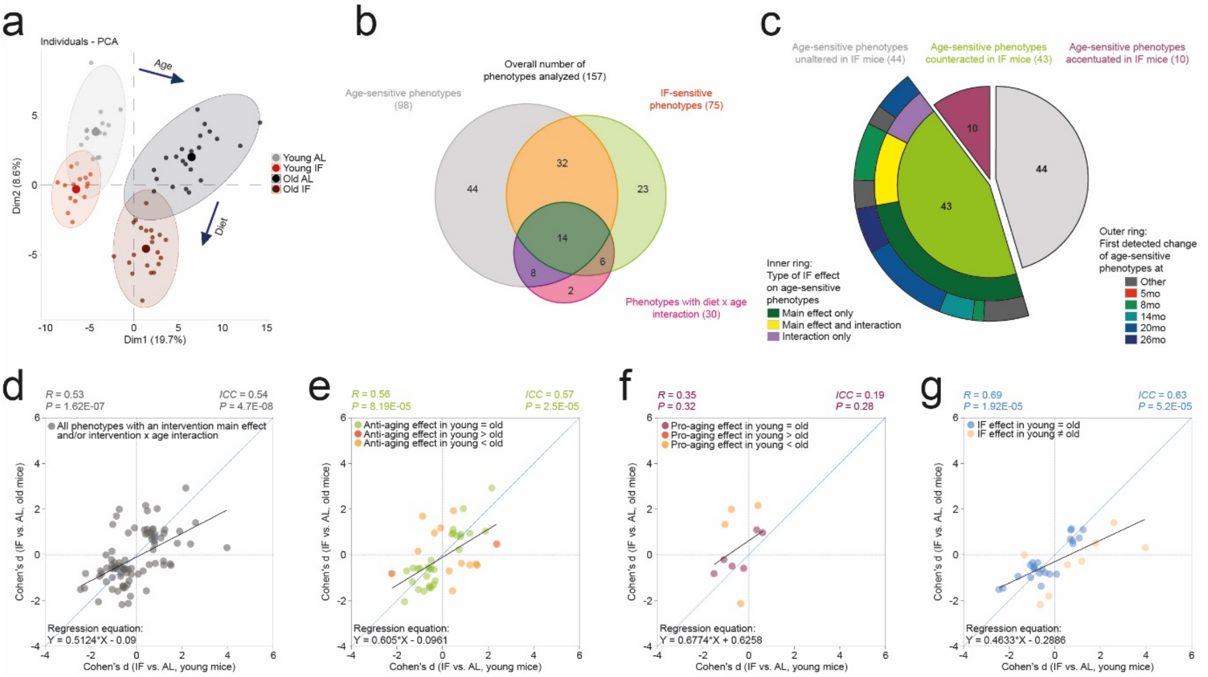

Supplementary Figure 16: Anti-aging effects induced by every-other-day fasting; analysis restricted to tumor-free mice. **a**, Principal component analysis of deep phenotyping data (number of mice: young AL, n=16; young IF, n=16; old AL, n=22; old IF, n=22). **b,** Venn diagram shows the number of age-sensitive phenotypes, diet-sensitive phenotypes, phenotypes with a diet × age interaction and their intersection. Two-way ANOVAs with the between-subjects factors age and diet followed by Fisher's LSD posthoc analyses (where appropriate) were used for data analysis. **c**, Sunburst chart shows the number of age-sensitive phenotypes either unaltered, counteracted or accentuated by IF. For age-sensitive phenotypes counteracted by IF, the inner ring shows the proportion of phenotypes with a main effect of diet, a diet × age interaction or both a main effect and an interaction. The outer ring shows when changes in the corresponding ASPs were first detected based on data available from our baseline study. **d**–**g**, Scatter plots show the effect size of IF in young mice plotted vs. the effect size of IF in old mice for different sets of phenotypes: **e**, ASPs counteracted by diet via a main effect and/or an interaction (i.e., corresponding to the central green section of the sunburst chart in **c**). **f**, ASPs accentuated by diet. **g**, Phenotypes featuring a main effect of diet and/or a diet × age interaction but not a main effect of age. **d**, all phenotypes shown in **e-g** collapsed into one panel. ICC = intraclass correlation. Statistical effect size comparisons were performed via two-sided z-tests. Our analyses are based on unadjusted p-values. For further details, see **Supplementary Data 9.**

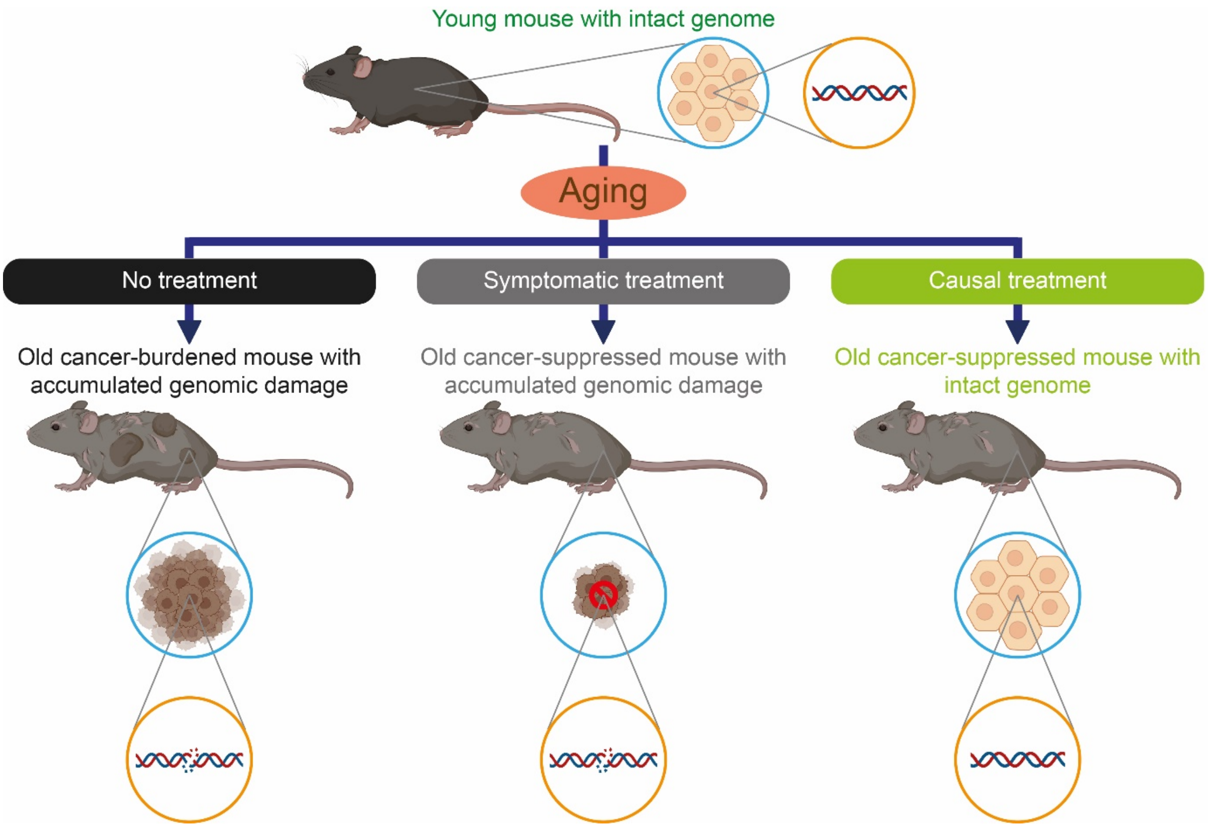

Supplementary Figure 17: Symptomatic and causal treatments can lead to the same outcome, but through different mechanisms. The differences between symptomatic and causal treatment are shown here using the age-related pathology "cancer" as an example. Under symptomatic treatment, tumor growth is blocked by non-specifically inhibiting cell proliferation via a cytostatic drug (middle lower panel). Importantly, however, the age-related accumulation of genome damage (that underlies cancer predisposition in old age in our example) remains unaffected by this type of approach. Causal treatment prevents the aging-associated accumulation of genome damage (right lower panel), thereby inhibiting cancer by targeting the biology underlying the age-related increase in cancer predisposition. Created with BioRender.com.

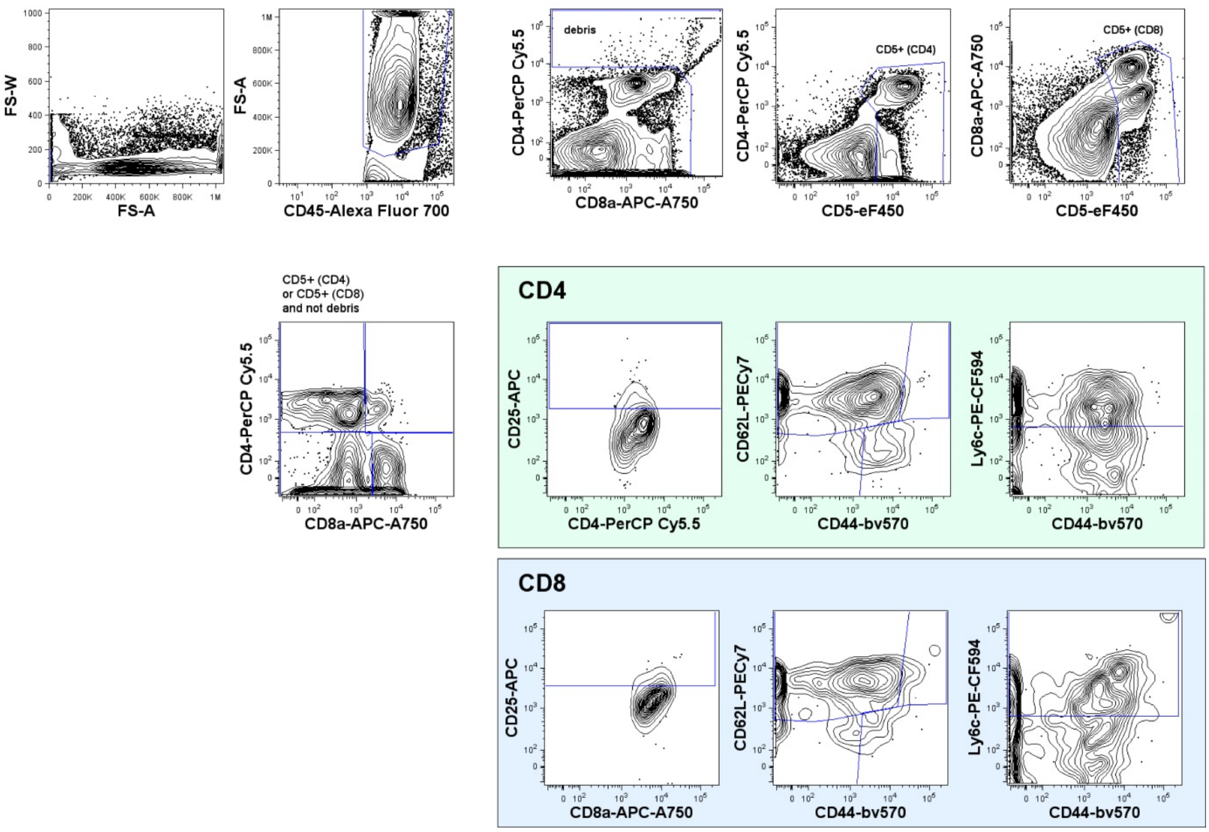

Supplementary Figure 18: Gating strategy of peripheral blood FACS analysis. We employed an acquisition threshold that facilitated the selective inclusion of CD45-positive leukocytes in the analysis. Surface antigens used to define major T cell populations and their subpopulations included CD4, CD5, CD8a, CD25, CD44, CD62L and Ly6C.

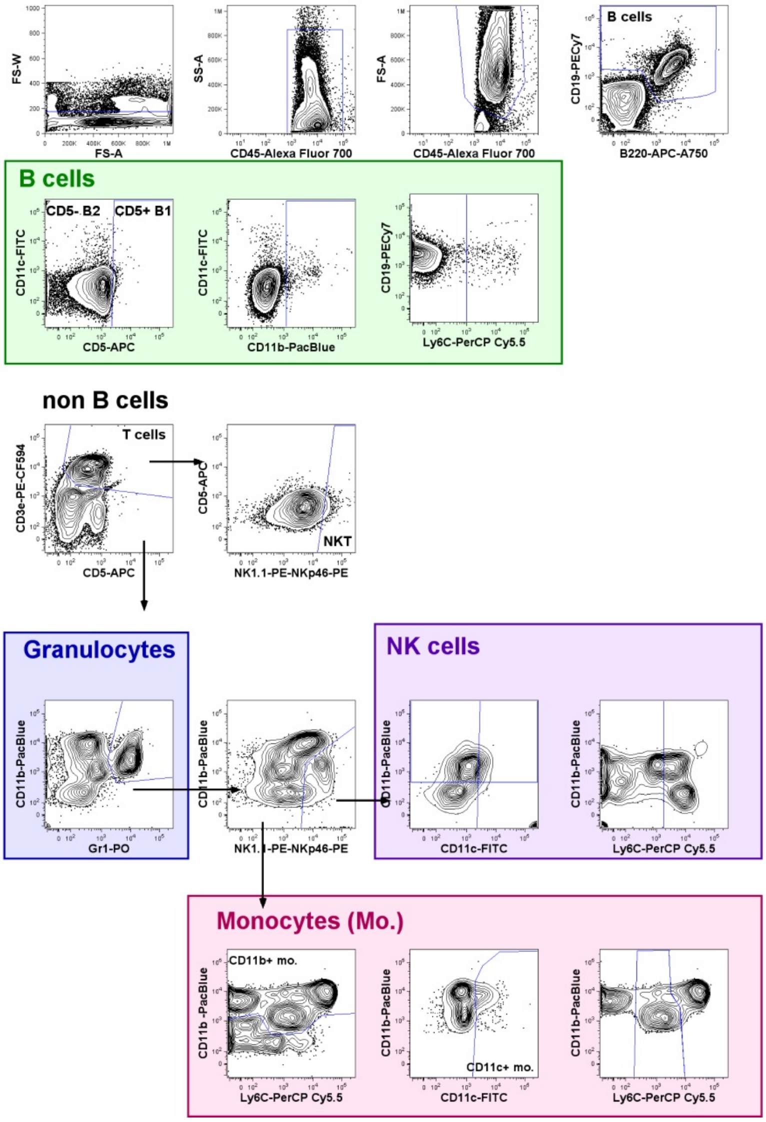

Supplementary Figure 19: Gating strategy of peripheral blood FACS analysis. We employed an acquisition threshold that facilitated the selective inclusion of CD45-positive leukocytes in the analysis. Surface antigens used to define B cell, granulocyte, monocyte and natural killer cell populations included B220, CD3e, CD5, CD11b, CD11c, CD19, Gr1, Ly6C, NK1.1 and NKp46.

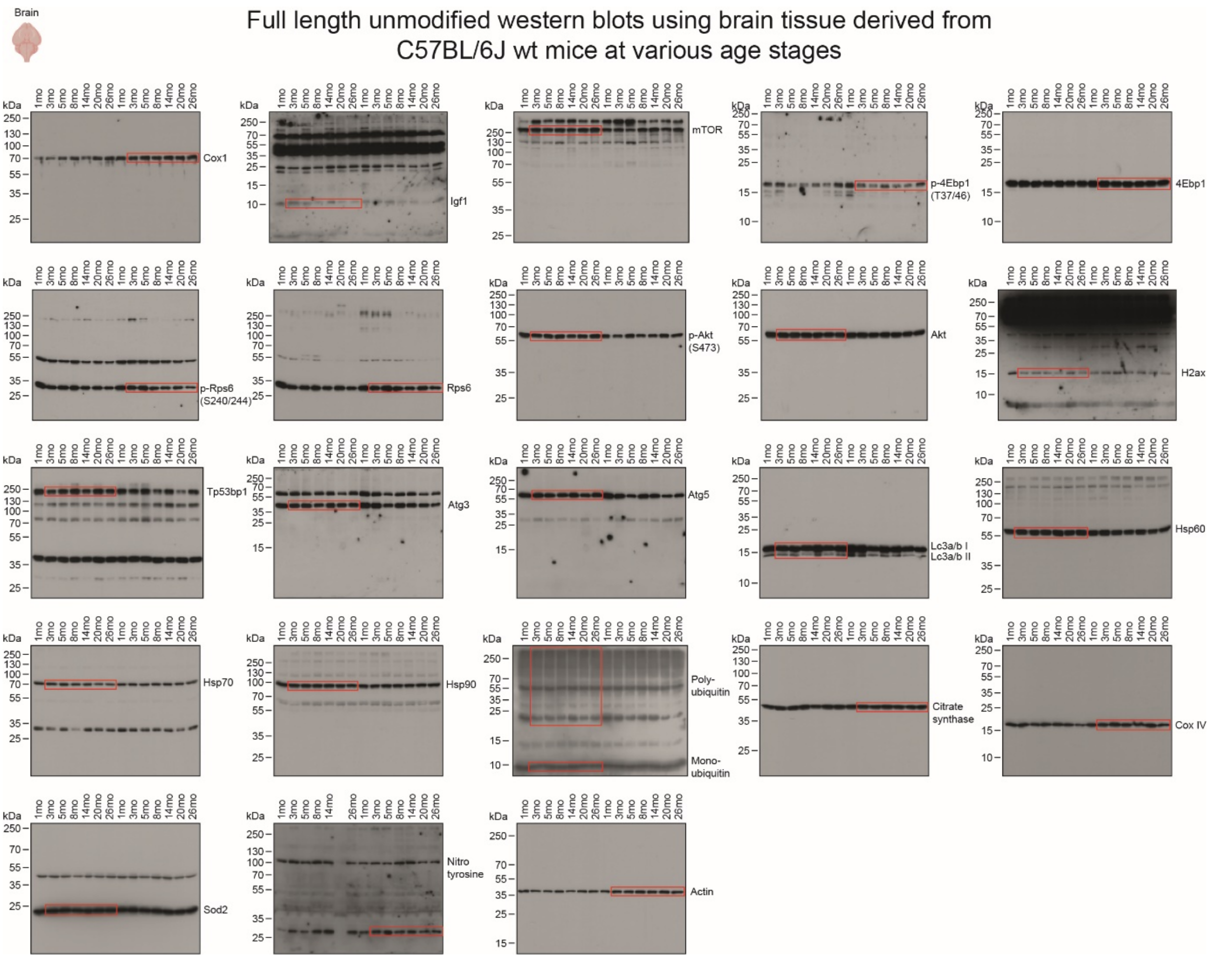

Supplementary Figure 20: Full length unmodified western blots performed using brain tissue derived from C57BL/6J wildtype mice at various ages. Representative western blots detecting Cox1, Igf1, mTor, p-4Ebp1(T37/46), total 4Ebp1, p-Rps6(S240/244), total Rps6, p-Akt(S473), total Akt, total H2ax, Tp53bp1, Atg3, Atg5, Lc3a/b, Hsp60, Hsp70, Hsp90, mono- and polyubiquitin, citrate synthase, Cox IV, Sod2, nitrotyrosine and actin are shown. 1mo = 1 month old; 3mo = 3 month old; 5mo = 5 month old; 8mo = 8month old; 14mo = 14 month old; 20mo = 20 month old; 26mo = 26 month old; kDa = kilodalton. Figure elements created with BioRender.com.

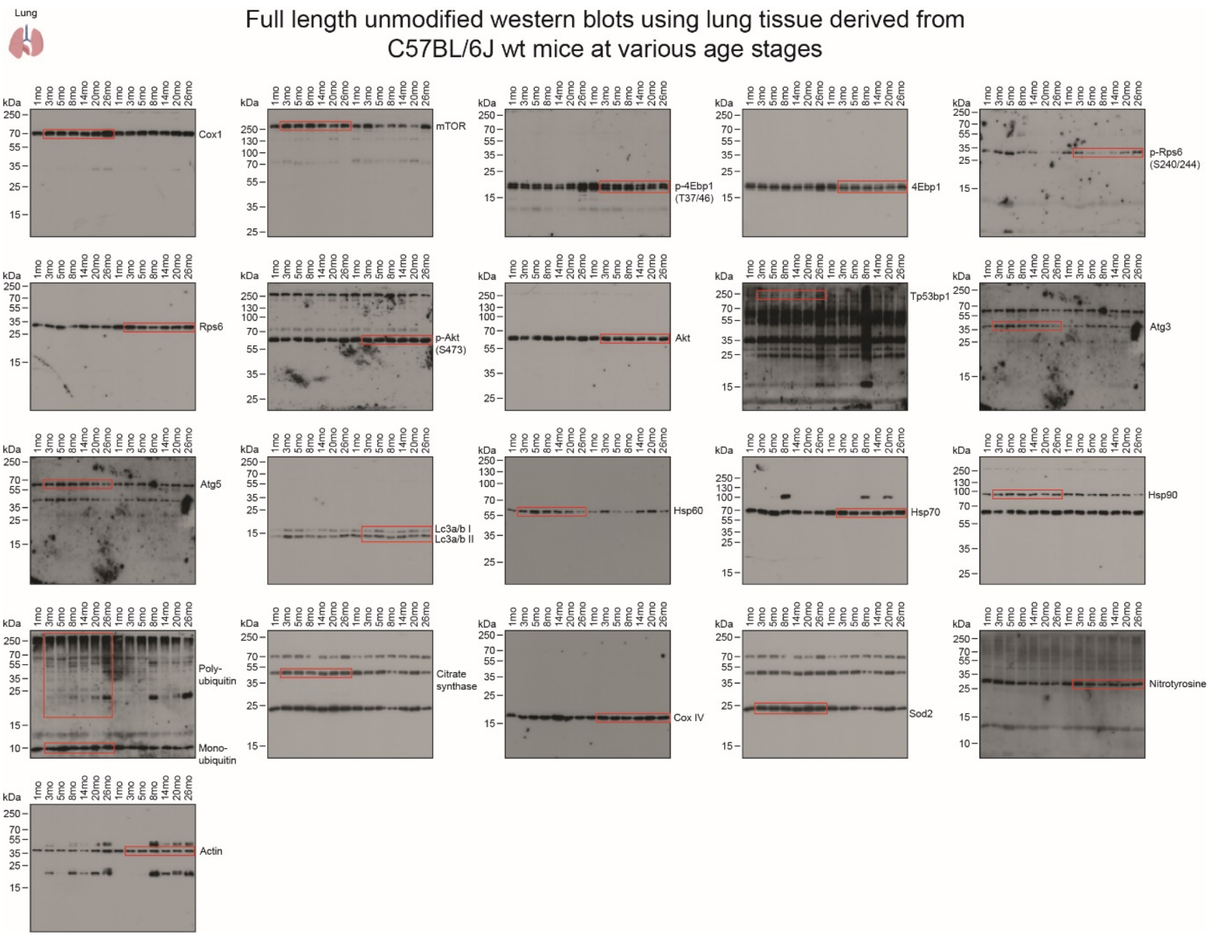

Supplementary Figure 21: Full length unmodified western blots performed using lung tissue derived from C57BL/6J wildtype mice at various ages. Representative western blots detecting Cox1, mTor, p-4Ebp1(T37/46), total 4Ebp1, p-Rps6(S240/244), total Rps6, p-Akt(S473), total Akt, Tp53bp1, Atg3, Atg5, Lc3a/b, Hsp60, Hsp70, Hsp90, mono- and polyubiquitin, citrate synthase, Cox IV, Sod2, nitrotyrosine and actin are shown. 1mo = 1 month old; 3mo = 3 month old; 5mo = 5 month old; 8mo = 8month old; 14mo = 14 month old; 20mo = 20 month old; 26mo = 26 month old; kDa = kilodalton. Figure elements created with BioRender.com.

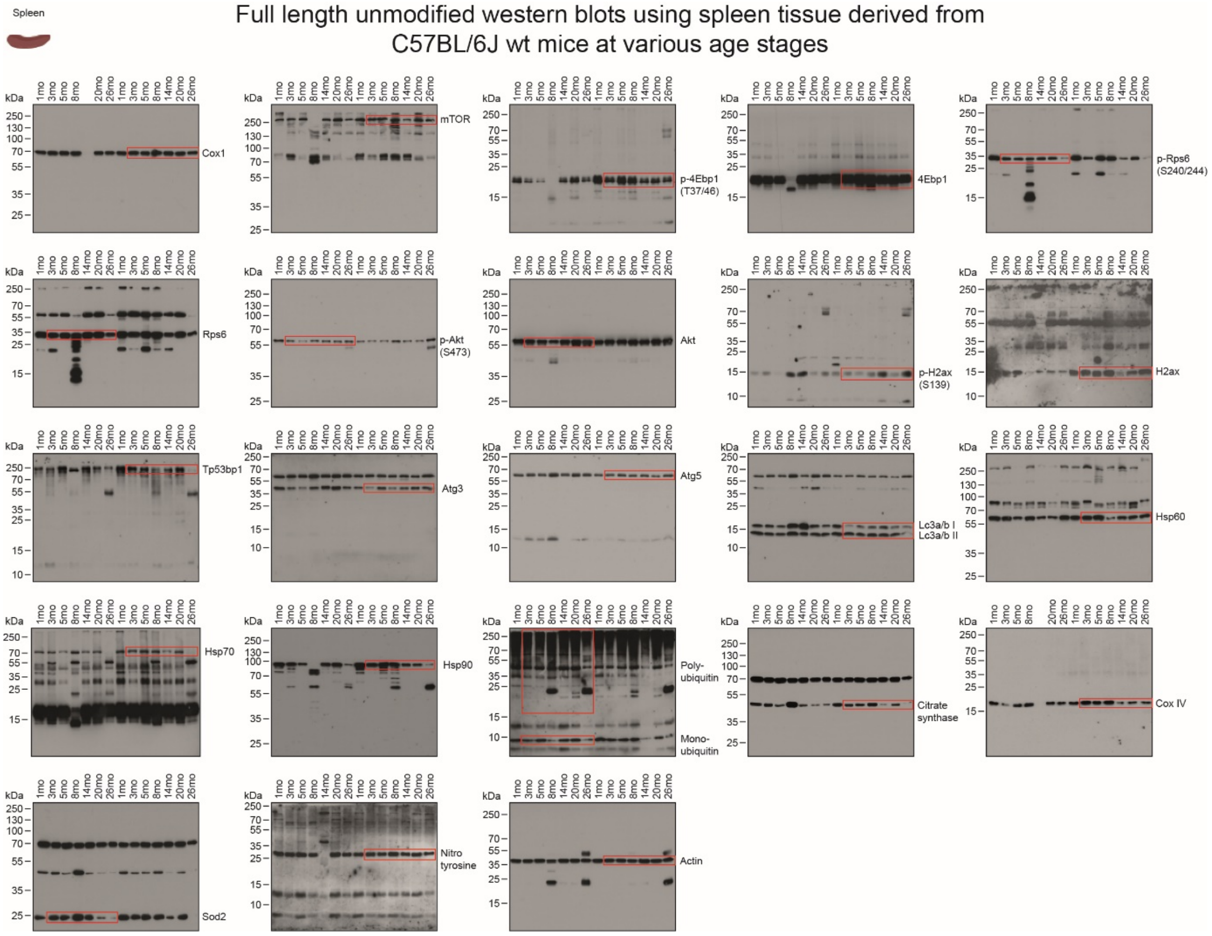

Supplementary Figure 22: Full length unmodified western blots performed using spleen tissue derived from C57BL/6J wildtype mice at various ages. Representative western blots detecting Cox1, mTor, p-4Ebp1(T37/46), total 4Ebp1, p-Rps6(S240/244), total Rps6, p-Akt(S473), total Akt, p-H2ax(S139), total H2ax, Tp53bp1, Atg3, Atg5, Lc3a/b, Hsp60, Hsp70, Hsp90, mono- and polyubiquitin, citrate synthase, Cox IV, Sod2, nitrotyrosine and actin are shown. 1mo = 1 month old; 3mo = 3 month old; 5mo = 5 month old; 8mo = 8month old; 14mo = 14 month old; 20mo = 20 month old; 26mo = 26 month old; kDa = kilodalton. Figure elements created with BioRender.com.

Supplementary References

1. Colao, A., *et al.* Bone loss is correlated to the severity of growth hormone deficiency in adult patients with hypopituitarism. *J Clin Endocrinol Metab* **84**, 1919-1924 (1999).
2. Leone, S., *et al.* Increased pain and inflammatory sensitivity in growth hormone-releasing hormone (GHRH) knockout mice. *Prostaglandins Other Lipid Mediat* **144**, 106362 (2019).
3. Berryman, D.E., *et al.* Two-year body composition analyses of long-lived GHR null mice. *J Gerontol A Biol Sci Med Sci* **65**, 31-40 (2010).
4. Leone, S., *et al.* Behavioural phenotyping, learning and memory in young and aged growth hormone-releasing hormone-knockout mice. *Endocr Connect* **7**, 924-931 (2018).
5. Wu, J.J., *et al.* Increased mammalian lifespan and a segmental and tissue-specific slowing of aging after genetic reduction of mTOR expression. *Cell Rep* **4**, 913-920 (2013).
6. Harrison, D.E., *et al.* Rapamycin fed late in life extends lifespan in genetically heterogeneous mice. *Nature* **460**, 392-395 (2009).
7. Miller, R.A., *et al.* Rapamycin, but not resveratrol or simvastatin, extends life span of genetically heterogeneous mice. *J Gerontol A Biol Sci Med Sci* **66**, 191-201 (2011).
8. Miller, R.A., *et al.* Rapamycin-Mediated Lifespan Increase in Mice is Dose and Sex-Dependent and Appears Metabolically Distinct from Dietary Restriction. *Aging Cell* **13**, 468-477 (2014).
9. Strong, R., *et al.* Rapamycin-mediated mouse lifespan extension: Late-life dosage regimes with sex-specific effects. *Aging Cell* **19**, e13269 (2020).
10. Neff, F., *et al.* Rapamycin extends murine lifespan but has limited effects on aging. *J Clin Invest* **123**, 3272-3291 (2013).
11. Wilkinson, J.E., *et al.* Rapamycin slows aging in mice. *Aging Cell* (2012).
12. Zhang, S., *et al.* Constitutive reductions in mTOR alter cell size, immune cell development, and antibody production. *Blood* **117**, 1228-1238 (2011).
13. Zhang, S., *et al.* B cell-specific deficiencies in mTOR limit humoral immune responses. *J Immunol* **191**, 1692-1703 (2013).
