# Supplementary material for: Deep phenotyping and lifetime trajectories reveal limited effects of longevity regulators on the aging process in C57BL/6J mice
Source: Nat Commun. 2022 Nov 11;13:6830. doi: 10.1038/s41467-022-34515-y (PMC9652467; doi:10.1038/s41467-022-34515-y)
Supplement: Supplementary file 2 — Description of Additional Supplementary Files [file 41467_2022_34515_MOESM2_ESM.pdf]

Description of Additional Supplementary Files

File Name: Supplementary Data 1
Description: summarizes the findings regarding our deep phenotyping analyses in C57BL/6J wildtype mice to identify age-sensitive phenotypes, including the age at first detected change relative to the young adult (3-month) baseline.

File Name: Supplementary Data 2
Description: provides details regarding sample sizes and summary statistics derived from our phenotypic analyses of 3-, 5-, 8-, 14-, 20- and 26-month old male C57BL/6J mice.

File Name: Supplementary Data 3
Description: shows results of differential expression analyses of spleen RNA-seq derived from 3-, 5-, 8-, 14-, 20- and 26-month old male C57BL/6J mice.

File Name: Supplementary Data 4
Description: shows results of differential expression analyses of brain RNA-seq derived from 3-, 5-, 8-, 14-, 20- and 26-month old male C57BL/6J mice.

File Name: Supplementary Data 5
Description: provides results of Ingenuity pathway analyses of the sets of genes differentially expressed, in either spleen or brain, in 5-, 8-, 14-, 20- or 26-month old male C57BL/6J mice relative to the 3-month old reference.

File Name: Supplementary Data 6
Description: provides details regarding sample sizes and summary statistics derived from our molecular analyses of 3-, 5-, 8-, 14-, 20- and 26-month old male C57BL/6J mice.

File Name: Supplementary Data 7
Description: summarizes age-sensitive phenotypes, derived from deep phenotyping analyses, countered or accentuated in *Ghrhr*$^{lit/lit}$ mutant mice.

File Name: Supplementary Data 8
Description: shows details regarding sample sizes and summary statistics derived from our phenotypic analyses of young and old *Ghrhr*$^{lit/lit}$ mutant mice and wildtype controls, respectively. We include two analyses, one focused on the complete set of mice and one including only tumor-free animals.

File Name: Supplementary Data 9
Description: summarizes age-sensitive phenotypes, derived from deep phenotyping analyses, countered or accentuated in *mTOR*$^{KI/KI}$ mutant mice.

File Name: Supplementary Data 10
Description: shows details regarding sample sizes and summary statistics derived from our phenotypic analyses of young and old *mTOR*$^{KI/KI}$ mutant mice and wildtype controls, respectively. We include two analyses, one focused on the complete set of mice and one including only tumor-free animals.

File Name: Supplementary Data 11
Description: shows results of differential expression analyses of RNA-seq data derived from young and old *mTOR*$^{KI/KI}$ mutant mice and wildtype controls, respectively.

File Name: Supplementary Data 12
Description: summarizes age-sensitive phenotypes, derived from deep phenotyping analyses, countered or accentuated in intermittent fasted mice.

File Name: Supplementary Data 13
Description: shows details regarding sample sizes and summary statistics derived from our phenotypic analyses of young and old mice subjected to intermittent fasting or ad libitum access to food. We include two analyses, one focused on the complete set of mice and one including only tumor-free animals.

File Name: Supplementary Data 14
Description: shows results of hierarchical clustering analyses of phenotypic data derived from young and old *Ghrhr*$^{lit/lit}$ mutant mice and wildtype controls, respectively. We include various analyses that are based on different cluster definitions (using different minimal inter-cluster distances).

File Name: Supplementary Data 15
Description: shows results of hierarchical clustering analyses of phenotypic data derived from young and old *mTOR*$^{KI/KI}$ mutant mice and wildtype controls, respectively. We include various analyses that are based on different cluster definitions (using different minimal inter-cluster distances).

File Name: Supplementary Data 16
Description: shows results of hierarchical clustering analyses of phenotypic data derived from young and old animals subjected to intermittent fasting or ad libitum access to food. We include various analyses that are based on different cluster definitions (using different minimal inter-cluster distances).
