# Supplementary material for: Deep phenotyping and lifetime trajectories reveal limited effects of longevity regulators on the aging process in C57BL/6J mice
Source: Nat Commun. 2022 Nov 11;13:6830. doi: 10.1038/s41467-022-34515-y (PMC9652467; doi:10.1038/s41467-022-34515-y)
Supplement: Supplementary file 19 — Reporting Summary [file 41467_2022_34515_MOESM19_ESM.pdf]

nature research

Corresponding author(s): Dan Ehninger

Last updated by author(s): Oct 8, 2022

Reporting Summary

Nature Research wishes to improve the reproducibility of the work that we publish. This form provides structure for consistency and transparency in reporting. For further information on Nature Research policies, see our Editorial Policies and the Editorial Policy Checklist.

Statistics

For all statistical analyses, confirm that the following items are present in the figure legend, table legend, main text, or Methods section.

n/a	Confirmed	
☐	☒	The exact sample size (*n*) for each experimental group/condition, given as a discrete number and unit of measurement
☐	☒	A statement on whether measurements were taken from distinct samples or whether the same sample was measured repeatedly
☐	☒	The statistical test(s) used AND whether they are one- or two-sided<br>*Only common tests should be described solely by name; describe more complex techniques in the Methods section.*
☐	☒	A description of all covariates tested
☐	☒	A description of any assumptions or corrections, such as tests of normality and adjustment for multiple comparisons
☐	☒	A full description of the statistical parameters including central tendency (e.g. means) or other basic estimates (e.g. regression coefficient) AND variation (e.g. standard deviation) or associated estimates of uncertainty (e.g. confidence intervals)
☐	☒	For null hypothesis testing, the test statistic (e.g. *F*, *t*, *r*) with confidence intervals, effect sizes, degrees of freedom and *P* value noted<br>*Give P values as exact values whenever suitable.*
☒	☐	For Bayesian analysis, information on the choice of priors and Markov chain Monte Carlo settings
☒	☐	For hierarchical and complex designs, identification of the appropriate level for tests and full reporting of outcomes
☐	☒	Estimates of effect sizes (e.g. Cohen's *d*, Pearson's *r*), indicating how they were calculated

Our web collection on statistics for biologists contains articles on many of the points above.

Software and code

Policy information about availability of computer code

Data collection	Data collection related to mouse phenotyping was completed by using custom made MausDB (open source, v1.5); Maier, H., Lengger, C., Simic, B.*, Fuchs, H., Gailus-Durner, V., Hrabě de Angelis, M.: MausDB: An open source application for phenotype data and mouse colony management in large-scale mouse phenotyping projects. BMC Bioinformatics 9:169 (2008)<br>Additional softwares applied included SkyScan volumetric NRecon reconstruction software (v1.7.5.0; Bruker, Billerica, MA, USA), CT analyser software (v1.15; Bruker, Billerica, MA, USA) and G*Power software (v3.1.9.2).
Data analysis	Data analyses were carried out using R version 3.5.1 and 3.6.1, including the packages FactoMineR_2.0, ggplot2_3.2.1, psych_2.0.7, ARTool_0.10.6, mice_3.13.0, effsize_0.7.6, car_3.0-5, Rmisc_1.5, agricolae_1.3-1, rcompanion_2.3.7, FSA_0.8.26, lattice_0.20-38, plyr_1.8.5, and GraphPad Prism version 8 (La Jolla, CA, US). Pathway enrichment analyses were performed using Ingenuity Pathway Analysis version 01-18-06 (Ingenuity Systems, Redwood City, CA, US). Analysis code is available at https://github.com/ehningerd/Xie_et_al-longevity_regulators. Prior to publication, the code is available for review via the following GitFront link: https://gitfront.io/r/user-4258314/80dd8f3e1e3778b5fa99aa93190e50020f64a23b/Xie-et-al_longevity-regulators/<br>Additional softwares used for data analysis included ImageJ (v1.50e; National Institute of Health), Flowjo (v10.8.1; TreeStar Inc, USA) and bclfastq2 (v2.20).

For manuscripts utilizing custom algorithms or software that are central to the research but not yet described in published literature, software must be made available to editors and reviewers. We strongly encourage code deposition in a community repository (e.g. GitHub). See the Nature Research guidelines for submitting code & software for further information.

Data

Policy information about availability of data

All manuscripts must include a data availability statement. This statement should provide the following information, where applicable:

- Accession codes, unique identifiers, or web links for publicly available datasets
- A list of figures that have associated raw data
- A description of any restrictions on data availability

Raw phenotypic and molecular data from Fig. 2, 3, 6 and 8 were deposited on Mendeley at https://data.mendeley.com/datasets/ypz9zyc9rp/draft?a=09b16f74-4581-48f7-94af-469e01757949. Raw sequencing data from Fig. 2, Supplementary Fig. 3 and 9 are available through GEO datasets at accession number GSE168068 (https://www.ncbi.nlm.nih.gov/geo/query/acc.cgi?acc=GSE168068). The Rhineland Study's dataset is not publicly available because of data protection regulations. Access to data can be provided to scientists in accordance with the Rhineland Study's Data Use and Access Policy. Requests for further information or to access the Rhineland Study's dataset should be directed to RS-DUAC@dzne.de.
The GTEx Portal (www.gtexportal.org, accessed on 05/30/20 and 07/15/20) was accessed to search for expression quantitative trait loci (eQTLs) related to the human GHRHR and MTOR genes.

Field-specific reporting

Please select the one below that is the best fit for your research. If you are not sure, read the appropriate sections before making your selection.

☒ Life sciences ☐ Behavioural & social sciences ☐ Ecological, evolutionary & environmental sciences

For a reference copy of the document with all sections, see nature.com/documents/nr-reporting-summary-flat.pdf

Life sciences study design

All studies must disclose on these points even when the disclosure is negative.

Sample size	Sample sizes of mouse cohorts were estimated using G*Power software (v3.1.9.2) as required for ethical approvals of animal experiments. In terms of clinical studies involving humans, assuming regression models with three predictors (i.e. age, genotype and an age × genotype interact term) and a type I error rate of 0.05, we estimated that with a sample size of ~3000 individuals we would be able to detect extremely small effect sizes of ≥ 0.0035 f2, defined according to Cohen (Cohen J., A power primer., Psychol. Bull 1992;112:155-159), with a statistical power of at least 80%.
Data exclusions	Not applicable since no samples or subjects were excluded
Replication	Experiments involving mouse or human subjects have been performed once, respectively.
Randomization	For our dietary intervention study, all animals were purchased as one cohort and were then randomly assigned to either the experimental or control group (intermittent fasting or ad libitum access to food).<br>For experiments involving Ghrhr and mTOR mutant mice lines, random allocation was not applicable. Ghrhr and mTOR mutant animals as well as their wild-type littermates entered the analysis pipeline after determination of their respective genotypes.<br>The human data presented derived from the Rhineland study, detailed described in the Methods section. Random allocation to experimental groups didn´t apply since all 3034 participants formed an entity to identify determinants and markers of healthy human aging.
Blinding	All experiments were conducted blind to study group assignment.

Reporting for specific materials, systems and methods

We require information from authors about some types of materials, experimental systems and methods used in many studies. Here, indicate whether each material, system or method listed is relevant to your study. If you are not sure if a list item applies to your research, read the appropriate section before selecting a response.

Materials & experimental systems

n/a	Involved in the study	
☐	☒	Antibodies
☒	☐	Eukaryotic cell lines
☒	☐	Palaeontology and archaeology
☐	☒	Animals and other organisms
☐	☒	Human research participants
☒	☐	Clinical data
☒	☐	Dual use research of concern

Methods

n/a	Involved in the study	
☒	☐	ChIP-seq
☐	☒	Flow cytometry
☒	☐	MRI-based neuroimaging

Antibodies

Antibodies used

All antibodies used are described in Extended Data Tables 2 and 5.

FITC-conjugated hamster anti-CD11c antibody; clone HL3; BD Pharmingen; #557400; species reactivity: human, mouse; validated applications: FC; dilution applied: 1:100; https://www.bdbiosciences.com/ko-kr/products/reagents/flow-cytometry-reagents/research-reagents/single-color-antibodies-ruo/fitc-hamster-anti-mouse-cd11c.557400

PE-conjugated mouse anti-NK1.1 antibody; cline PK136; BD Pharmingen; #553165; species reactivity: mnouse; validated applications: FC; dilution applied: 1:200; https://www.bdbiosciences.com/en-nz/products/reagents/flow-cytometry-reagents/research-reagents/single-color-antibodies-ruo/pe-mouse-anti-mouse-nk-1-1.553165#

PE-conjugated rat anti-NKp46 antibody; clone 29A1.4; eBioscience; #12-3351-82; species reactivity: mouse; validated applications: ICC, FC; dilution applied: 1:200; https://www.thermofisher.com/antibody/product/CD335-NKp46-Antibody-clone-29A1-4-Monoclonal/12-3351-82

PE-CF594-conjugated hamster anti-CD3e antibody; clone 145-2C11; BD Horizon; #562332; species reactivity: mouse; validated applications: FC; dilution applied: 1:100; https://www.bdbiosciences.com/en-nz/products/reagents/flow-cytometry-reagents/research-reagents/single-color-antibodies-ruo/pe-cf594-hamster-anti-mouse-cd3e.562332

PerCP Cy5.5-conjugated rat anti-Ly6C antibody; cloneHK1.4; eBioscience; #45-5932-82; species reactivity: mouse; validated applications: FC; dilution applied: 1:400; https://www.bdbiosciences.com/en-nz/products/reagents/flow-cytometry-reagents/research-reagents/single-color-antibodies-ruo/pe-cy-7-rat-anti-mouse-cd19.552854

PECy7-conjugated rat anti-CD19 antibody; clone 1D3; BD Pharmingen; #552854; species reactivity: mouse; validated applications: FC; dilution applied: 1:1000; https://www.bdbiosciences.com/en-nz/products/reagents/flow-cytometry-reagents/research-reagents/single-color-antibodies-ruo/pe-cy-7-rat-anti-mouse-cd19.552854

APC-conjugated rat anti-CD5 antibody; clone 53-7.3; BD Pharmingen; #550035; species reactivity: mouse; validated applications: FC; dilution applied: 1:2000; https://www.bdbiosciences.com/en-nz/products/reagents/flow-cytometry-reagents/research-reagents/single-color-antibodies-ruo/apc-rat-anti-mouse-cd5.550035

Alexa Fluor 700-conjugated rat anti-CD45 antibody; clone 30-F11; BioLegend; #103128; species reactivity: mouse; validated applications: FC; dilution applied: 1:1000; https://www.biolegend.com/en-us/products/alexa-fluor-700-anti-mouse-cd45-antibody-3407

APC-A750-conjugated rat anti-B220 antibody; clone RA3-6B2; Life Technologies; #RM2627; species reactivity: mouse; validated applications: FC; dilution applied: 1:100; https://www.thermofisher.com/antibody/product/CD45R-Antibody-clone-RA3-6B2-Monoclonal/RM2627

PacBlue-conjugated rat anti-CD11b antibody; clone M1/70.15; Life Technologies; #RM2828; species reactivity: mouse; validated applications: FC; dilution applied: 1:800; https://www.thermofisher.com/antibody/product/CD11b-Antibody-clone-M1-70-15-Monoclonal/RM2828

PO-conjugated rat anti-Gr1 antibody; clone RB6-8C5; Life Technologies; #RM3030; species reactivity: mouse; validated applications: IHC, FC; dilution applied: 1:1000; https://www.thermofisher.com/antibody/product/Ly-6G-Ly-6C-Antibody-clone-RB6-8C5-Monoclonal/RM3030

PE-CF594-conjugated rat anti-Ly6C antibody; clone AL-21; BD Horizon; #562728; species reactivity: mouse; validated applications: FC; dilution applied: 1:200; https://www.bdbiosciences.com/en-nz/products/reagents/flow-cytometry-reagents/research-reagents/single-color-antibodies-ruo/pe-cf594-rat-anti-mouse-ly-6c.562728

PerCP Cy5.5-conjugated rat anti-CD4 antibody; clone RM4-5; Tonbo Biosciences; #65-0042-U025; species reactivity: mouse; validated applications: FC; dilution applied: 1:1000; https://tonbobio.com/products/percp-cyanine5-5-anti-mouse-cd4-rm4-5

PECy7-conjugated rat anti-CD62L antibody; clone MEL-14; eBioscience; #25-0621-82; species reactivity: mouse; validated applications: FC; dilution applied: 1:2000; https://www.bdbiosciences.com/en-nz/products/reagents/flow-cytometry-reagents/research-reagents/single-color-antibodies-ruo/apc-rat-anti-mouse-cd25.557192

APC-conjugated rat anti-CD25 antibody; clone PC61; BD Pharmingen; #557192; species reactivity: mouse; validated applications: FC; dilution applied: 1:100; https://www.bdbiosciences.com/en-nz/products/reagents/flow-cytometry-reagents/research-reagents/single-color-antibodies-ruo/apc-rat-anti-mouse-cd25.557192

APC-A750-conjugated rat anti-CD8a antibody; clone 5H10; Life Technologies; #MCD0827; species reactivity: mouse; validated applications: FC; dilution applied: 1:400; https://www.thermofisher.com/antibody/product/CD8-alpha-Antibody-clone-5H10-Monoclonal/MCD0827

eF450-conjugated rat anti-CD5 antibody; clone 53-7.3; eBioscience; #48-0051-82; species reactivity: mouse; validated applications: FC; dilution applied: 1:1000; https://www.thermofisher.com/antibody/product/CD5-Antibody-clone-53-7-3-Monoclonal/48-0051-82

bv570-conjugated rat anti-CD44 antibody; clone IM7; BioLegend; #103037; species reactivity: mouse; validated applications: FC; dilution applied: 1:100; https://www.biolegend.com/en-ie/products/brilliant-violet-570-anti-mouse-human-cd44-antibody-7386

rat anti-CD3 antibody; clone 17A2; eBioscience; #16-0032-86; species reactivity: mouse; validated application: WB, IHC, ICC, FC;

dilution applied: 1 μg/ml; https://www.thermofisher.com/antibody/product/CD3-Antibody-clone-17A2-Monoclonal/16-0032-86

hamster anti-CD40 antibody; clone HM40-3; eBiocience; #16-0402-86; species reactivity: mouse, rat; validated applications: WB, FC; dilution applied: 1 μg/ml; https://www.thermofisher.com/antibody/product/CD40-Antibody-clone-HM40-3-Monoclonal/16-0402-86

rabbit anti-Cox1 antibody; polyclonal; Cell Signaling Technologies; #4841; species reactivity: human, mouse, rat; validated applications: WB, FC; dilution applied: 1:2000; https://www.cellsignal.com/products/primary-antibodies/cox1-antibody/4841

rabbit anti-Igf1 antibody; polyclonal; Abcam; #ab9572; species reactivity: mouse, human; validated applications: ICC, IHC, WB, ELISA; dilution applied: 1:1000; https://www.abcam.com/igf1-antibody-ab9572.html

rabbit anti-mTOR antibody; clone 7C10; Cell Signaling Technologies; #2983; species reactivity: human, mouse, rat, monkey; validated applications: WB, IP, IHC, ICC, FC; dilution applied: 1:2000; https://www.cellsignal.com/products/primary-antibodies/mtor-7c10-rabbit-mab/2983

rabbit anti-p-4Ebp1 (T37/46) antibody; clone 236B4; Cell Signaling Technologies; #2855; species reactivity: human, mouse, rat, monkey, D. melanogaster; validated applications: WB, IHC, ICC, FC; dilution applied: 1:2000; https://www.cellsignal.com/products/primary-antibodies/phospho-4e-bp1-thr37-46-236b4-rabbit-mab/2855

rabbit anti-4Ebp1 antibody; clone 53H11; Cell Signaling Technologies; #9644; species reactivity: human, mouse, rat, monkey; validated applications: WB, IP, IHC, ICC, FC; dilution applied: 1:30000; https://www.cellsignal.com/products/primary-antibodies/4e-bp1-53h11-rabbit-mab/9644

rabbit anti-p-Rps6 (S240/244) antibody; polyclonal; Cell Signaling Technologies; #2215; species reactivity: human, mouse, rat, monkey, zebrafish; validated applications: WB, IP; dilution applied: 1:2000; https://www.cellsignal.com/products/primary-antibodies/phospho-s6-ribosomal-protein-ser240-244-antibody/2215

rabbit anti-Rps6 antibody; clone 5G10; Cell Signaling Technologies; #2217; species reactivity: human, mouse, rat, monkey; validated applications: WB, IHC, ICC; dilution applied: 1:10000; https://www.cellsignal.com/products/primary-antibodies/s6-ribosomal-protein-5g10-rabbit-mab/2217

rabbit anti-p-Akt (S473) antibody; polyclonal; Cell Signaling Technologies; #9271; species reactivity: human, mouse, rat, hamster, monkey, D. melanogaster, bovine, dog; validated applications: WB, IP, ICC, FC; dilution applied: 1:2000; https://www.cellsignal.com/products/primary-antibodies/phospho-akt-ser473-antibody/9271

rabbit anti-Akt; polyclonal antibody; Cell Signaling Technologies; #9272; species reactivity: human, mouse, rat, hamster, monkey, chicken, D. melanogaster, bovine, dog, pig, guinea pig; validated applications: WB, IP, IHC, FC; dilution applied: 1:5000; https://www.cellsignal.com/products/primary-antibodies/akt-antibody/9272

rabbit anti-p-H2ax (S139) antibody; polyclonal; Cell Signaling Technologies; #2577; species reactivity: human, mouse, rat, monkey; validated applications: WB, IHC, FC; dilution applied: 1:2000; https://www.cellsignal.com/products/primary-antibodies/phospho-histone-h2a-x-ser139-antibody/2577

rabbit anti-H2ax antibody; polyclonal; Cell Signaling Technologies; #2595; species reactivity: human, mouse, rat, monkey; validated applications: WB; dilution applied: 1:2000; https://www.cellsignal.com/products/primary-antibodies/histone-h2a-x-antibody/2595

rabbit anti-Tp53bp1 antibody; polyclonal; Abnova; #PAB12506; species reactivity: human, mouse; validated applications: ICC, IP, WB; dilution applied: 1:2000; https://www.abnova.com/products/products_detail.asp?Catalog_id=PAB12506

rabbit anti-Atg3 antibody; polyclonal; Cell Signaling Technologies; #3415; species reactivity: human, mouse, rat, monkey; validated applications: WB; dilution applied: 1:2000; https://www.cellsignal.com/products/primary-antibodies/atg3-antibody/3415

rabbit anti-Atg5 antibody; clone D5F5U; Cell Signaling Technologies; #12994; species reactivity: human, mouse, rat; validated applications: WB, IP; dilution applied: 1:2000; https://www.cellsignal.com/products/primary-antibodies/atg5-d5f5u-rabbit-mab/12994

rabbit anti-Lc3a/b antibody; clone D3U4C; Cell Signaling Technologies; #12741; species reactivity: human, mouse, rat; validated applications: WB, IHC, ICC, FC; dilution applied: 1:3000; https://www.cellsignal.com/products/primary-antibodies/lc3a-b-d3u4c-xp-rabbit-mab/12741

rabbit anti-Hsp60 antibody; clone D307; Cell Signaling Technologies; #4870; species reactivity: human, mouse, rat, monkey, D. melanogaster; validated applications: WB, ICC, FC; dilution applied: 1:10000; https://www.cellsignal.com/products/primary-antibodies/hsp60-d307-antibody/4870

rabbit anti-Hsp70 antibody; polyclonal; Cell Signaling Technologies; #4872; species reactivity: human, mouse, rat, monkey, bovine; validated applications: WB, IHC; dilution applied: 1:10000; https://www.cellsignal.com/products/primary-antibodies/hsp70-antibody/4872

rabbit anti-Hsp90 antibody; polyclonal; Cell Signaling Technologies; #4874; species reactivity: human, mouse, rat, monkey, D. melanogaster, zebrafish; validated applications: WB, IHC; dilution applied: 1:10000; https://www.cellsignal.com/products/primary-antibodies/hsp90-antibody/4874

rabbit anti-Ubiquitin antibody; polyclonal; Thermo Fisher Scientific; #PA5-76144; species reactivity: human, mouse rat; validated applications: WB, IHC, ICC; dilution applied: 1:2000; https://www.thermofisher.com/antibody/product/Ubiquitin-C-Antibody-Polyclonal/PA5-76144

rabbit anti-citrate synthase antibody; clone D7V8B; Cell Signaling Technologies; #14309; species reactivity: human, mouse, rat, hamster, monkey; validated applications: WB, ICC; dilution applied: 1:2000; https://www.cellsignal.com/products/primary-antibodies/citrate-synthase-d7v8b-rabbit-mab/14309

rabbit anti-Cox IV antibody; clone 3E11; Cell Signaling Technologies; #4850; species reactivity: human, rat, monkey, zebrafish, bovine, pig; validated applications: WB, IP, IHC, ICC, FC; dilution applied: 1:2000; https://www.cellsignal.com/products/primary-antibodies/cox-iv-3e11-rabbit-mab/4850

rabbit anti-Sod2 antibody; clone D9V9C; Cell Signaling Technologies; #13194; species reactivity: human, mouse, rat, monkey; validated applications: WB; dilution applied: 1:2000; https://www.cellsignal.com/products/primary-antibodies/sod2-d9v9c-rabbit-mab/13194

rabbit anti-nitrotyrosine; Enzo Life Science; #BML-SA297-0100; species reactivity: species independent; validated applications: ELISA, WB; dilution applied: 1:2000; https://www.enzolifesciences.com/BML-SA297/nitrotyrosine-polyclonal-antibody/

mouse anti-actin antibody; clone C4; MP Biomedicals; #SKU 0869100; species reactivity: human, mouse, rat, guinea pig; validated applications: ELISA, IHC, WB, ICC; dilution applied: 1:30000; https://www.mpbio.com/bs/anti-actin-mouse-monoclonal-antibody-clone-c4

horseradish peroxidase-conjugated goat anti rabbit antibody; polyclonal; Promega; #W4011; species reactivity: rabbit; validated applications: WB, ELISA; dilution applied: 1:3000; https://www.promega.de/products/protein-detection/primary-and-secondary-antibodies/anti-rabbit-igg-h-and-l-hrp-conjugate/?catNum=W4011#specifications

horseradish peroxidase-conjugated goat anti mouse antibody; polyclonal; Agilent Technologies; #P0447; species reactivity: mouse; dilution applied: 1:10000; https://www.agilent.com/en/product/specific-proteins/elisa-kits-accessories/goat-anti-mouse-immunoglobulins-hrp-affinity-isolated-2717109

Abbreviations: ELISA = Enzyme-linked immunosorbent assay; FC = Flow cytometry; ICC = Immunocytochemistry; IHC = Immunohistochemistry; IP = Immunoprecipitation; WB = Western Blot

Validation

All antibodies applied have been validated by the manufacturer for the given application. Manufacturer´s instructions in terms of recommended dilutions have been followed.

Animals and other organisms

Policy information about studies involving animals; ARRIVE guidelines recommended for reporting animal research

Laboratory animals

All animal experiments described were limited to male mice only. Wild-type C57BL/6J mice (3 months, 5 months, 8 months, 14 months, 20 months and 26 months of age) purchased from Charles River Laboratories were used to determine the trajectories of aging-sensitive phenotypes in mice. Three-longevity mouse models were included in our analyses to estimate whether longevity may be associated with a general retardation of aging in mice. They featured either a loss-of-function mutation in the Ghrhr gene (Ghrhrlit mutation), carried a hypomorphic mTOR mutant allele or were subjected to almost lifelong intermittent fasting.

Wild animals

Study did not involve wild animals

Field-collected samples

Study did not involve samples collected in the field

Ethics oversight

In accordance with the German Animal Welfare Act, studies related to the mouse was approved by the "Landesamt für Natur, Umwelt und Verbraucherschutz Nordrhein-Westfalen" (Recklinghausen, Germany) as well as the "Regierung von Oberbayern" (Munich, Germany).

Note that full information on the approval of the study protocol must also be provided in the manuscript.

Human research participants

Policy information about studies involving human research participants

Population characteristics

The population is described in Extended Data Table 8

Recruitment

The human data were collected in the context of the Rhineland Study, which is an ongoing, large-scale, single-center, population-based prospective cohort study among people aged 30 years and above in Bonn, Germany. The only exclusion criterion is insufficient command of the German language to provide informed consent.

Ethics oversight

The study was approved by the ethics committee of the Medical Faculty of the University of Bonn.

Note that full information on the approval of the study protocol must also be provided in the manuscript.

Flow Cytometry

Plots

Confirm that:

☒ The axis labels state the marker and fluorochrome used (e.g. CD4-FITC).

☒ The axis scales are clearly visible. Include numbers along axes only for bottom left plot of group (a 'group' is an analysis of identical markers).

☒ All plots are contour plots with outliers or pseudocolor plots.

☒ A numerical value for number of cells or percentage (with statistics) is provided.

Methodology

Sample preparation	Whole blood samples were incubated with Fc block (clone 2.4G2) for 5 min at 4–10°C. Subsequently, blood leukocytes were stained using a mixture of fluorescence-conjugated monoclonal antibodies (BD Biosciences, Franklin Lakes, NJ, US) for 1h at 4–10°C. Prior to the analysis of samples, we carried out lysis of erythrocytes and applied formalin-based fixation. Detailed information regarding the definition of relevant cell subpopulations and gating strategies applied is provided in Supplementary Table 1 as well as Supplementary Fig. 18 and 19.
Instrument	Samples were analyzed using a Gallios ten-color flow cytometer (Beckman Coulter, Brea, CA, US) combined with an IntelliCyt HyperCyt sampler (Sartorius, Göttingen, Germany)
Software	Frequencies of leukocyte populations were determined by software-based analysis (Flowjo, v10.8.1, TreeStar Inc, USA; and SPICE; Roederer, M., Nozzi, J. L. & Nason, M. C. SPICE: exploration and analysis of post-cytometric complex multivariate datasets. Cytometry A 79, 167-174, doi:10.1002/cyto.a.21015 (2011))
Cell population abundance	A minimum of 10,000/Leukocytes or 2000/Lymphocytes per sample was examined.
Gating strategy	Gates for each parameter were based on formerly performed 'fluorescence minus one' (FMO) controls. Surface antigens used to define leukocyte populations were: B220, CD3, CD4, CD5, CD8, CD11b, CD11c, CD19, CD25, CD44, CD62L, Ly6C, Ly6G, NK1.1, NKp46 and gamma delta T cell receptor (gdTCR). Detailed information regarding the definition of relevant cell subpopulations and gating strategies applied is provided in Supplementary Table 1 as well as Supplementary Fig. 18 and 19.

☒ Tick this box to confirm that a figure exemplifying the gating strategy is provided in the Supplementary Information.
